# Monocyte inflammation and adaptive immune dysfunction in severe *Klebsiella pneumoniae* pneumonia

**DOI:** 10.1002/imt2.70172

**Published:** 2026-09-21

**Authors:** Hongquan Chen, Xiaoling Wang, Xiang Liu, Kui Dong, Yanying Feng, Min Yuan, Shiyi Wang, Fangfang Fan, Yong Tian, Zhiming Kang, Xiushuai Du, Chaolu Hasi, Hong Lu, Wei Guan, Xu Chen, Mengyu Cheng, Wei Wei, Nan Guo, Zhiguo Liu, Xiaotong Qiu, Yiwei Shi, Kun Xiao, Yi Wang, Zhenjun Li

**Affiliations:** ^1^ National Key Laboratory of Intelligent Tracking and Forecasting for Infectious Diseases, National Institute for Communicable Disease Control and Prevention Chinese Center for Disease Control and Prevention & Chinese Academy of Preventive Medicine Beijing China; ^2^ Department of Clinical Laboratory Shanxi Hospital of Traditional Chinese Medicine Taiyuan China; ^3^ NHC Key Laboratory of Pneumoconiosis, MOE Key Laboratory of Coal Environmental Pathogenicity and Prevention, Shanxi Key Laboratory of Chronic Respiratory Diseases and Pneumoconiosis The First Hospital of Shanxi Medical University Taiyuan Shanxi China; ^4^ Research Center for Reverse Microbial Etiology, Workstation of Academician Shanxi Medical University Taiyuan People's Republic of China; ^5^ Ophthalmic Microbiology Laboratory, Shanxi Eye Hospital Shanxi Medical University Taiyuan Shanxi People's Republic of China; ^6^ Department of Internal Medicine, The First Clinical Medical College Shanxi Medical University Taiyuan Shanxi China; ^7^ School of Public Health, Key Lab of Public Health Safety of the Ministry of Education and NHC Key Laboratory of Health Technology Assessment Fudan University Shanghai China; ^8^ Department of Laboratory Medicine First Hospital of Shanxi Medical University 85 Jiefang South Road Taiyuan Shanxi China; ^9^ Department of Respiratory and Critical Care Medicine Shanxi Hospital of Traditional Chinese Medicine Taiyuan China; ^10^ Department of Respiratory and Critical Care Medicine, Shanxi Bethune Hospital, Shanxi Academy of Medical Sciences, Tongji Shanxi Hospital Third Hospital of Shanxi Medical University Taiyuan China; ^11^ College of Pulmonary & Critical Care Medicine Chinese PLA General Hospital Beijing People's Republic of China; ^12^ Experimental Research Center & Molecular Diagnostic Center, Capital Center for Children's Health Capital Medical University, Capital Institute of Pediatrics Beijing People's Republic of China; ^13^ Beijing Research Center for Respiratory Infectious Diseases Beijing China

**Keywords:** adaptive immune dysfunction, inflammatory amplification, *Klebsiella pneumoniae* pneumonia, peripheral immune landscape, single‐cell RNA sequencing

## Abstract

Severe *Klebsiella pneumoniae* pneumonia is associated with substantial mortality and is characterized by intense systemic inflammation that coexists with a failure to control infection. The cellular changes underlying the transition from effective host defense to immune dysfunction remain incompletely defined. Here, we constructed a severity‐focused single‐cell transcriptomic atlas of 708,894 peripheral blood mononuclear cells from 100 individuals (54 healthy controls, 23 patients with mild disease, and 23 patients with severe disease), complemented by plasma protein measurements and flow cytometry. Mild disease was characterized by a relatively coordinated peripheral immune response, with preserved monocyte regulatory and tissue‐repair programs, preserved cytotoxic lymphocyte activity, and coordinated interactions between T follicular helper cells and B cells. Severe disease showed marked monocyte inflammation together with widespread disruption of adaptive immune responses. Classical monocytes expanded and showed increased expression of *S100A8/A9/A12*, accompanied by enhanced *TLR4–MYD88* signaling and elevated plasma S100 proteins. These changes were accompanied by impaired dendritic cell antigen presentation, expansion of immunoregulatory monocyte states resembling monocytic myeloid‐derived suppressor cells, cytotoxicity‐ and exhaustion‐associated changes in CD8^+^ T cells and natural killer cells, hyperactivated yet poorly coordinated CD4^+^ T cell states, and weaker B cell receptor signaling despite plasma cell expansion. These findings delineate a severity‐associated peripheral immune state characterized by monocyte‐centered inflammatory signatures and features of altered adaptive immune coordination, and provide a basis for future mechanistic investigation of monocyte‐derived *S100* alarmins and severity‐associated myeloid remodeling.

## Highlights

- *Klebsiella pneumoniae* pneumonia severity maps to distinct peripheral immune states, with relatively coordinated responses in mild disease and altered immune coordination in severe disease.
- *S100A8/A9/A12*‐high classical monocytes characterize the inflammatory landscape of severe disease.
- Severe disease features immunoregulatory monocyte expansion and reduced dendritic cell antigen presentation programs.
- CD8/NK and CD4^+^ T cell remodeling accompanies altered adaptive immune coordination in severe disease.

## Graphical abstract

Severe *Klebsiella pneumoniae* pneumonia combines intense inflammation with failure to control infection, yet the cellular basis is unclear. Using a severity‐focused single‐cell atlas of 708,894 peripheral blood mononuclear cells from 100 individuals, complemented by plasma protein measurements and flow cytometry, we show that disease severity redirects rather than simply amplifies systemic immunity. Mild disease preserves coordinated host defense: immune‐regulatory and tissue‐reparative monocytes, intact cytotoxic lymphocytes, and T follicular helper–B cell coordination. Severe disease is characterized by *S100A8/A9/A12*‐high inflammatory monocytes with enhanced *TLR4–MYD88* signaling, expansion of monocytic myeloid‐derived suppressor cell‐like states, and impaired dendritic cell antigen presentation. These myeloid changes accompany dysfunctional CD4^+^ T cells, cytotoxicity‐ and exhaustion‐associated CD8/NK cells, and weakened expression of B cell receptor signaling‐related genes despite plasma cell accumulation. Severe disease is characterized by a systemic pattern of impaired immune coordination, highlighting monocyte‐derived *S100* alarmins as candidates for future mechanistic and therapeutic investigation.

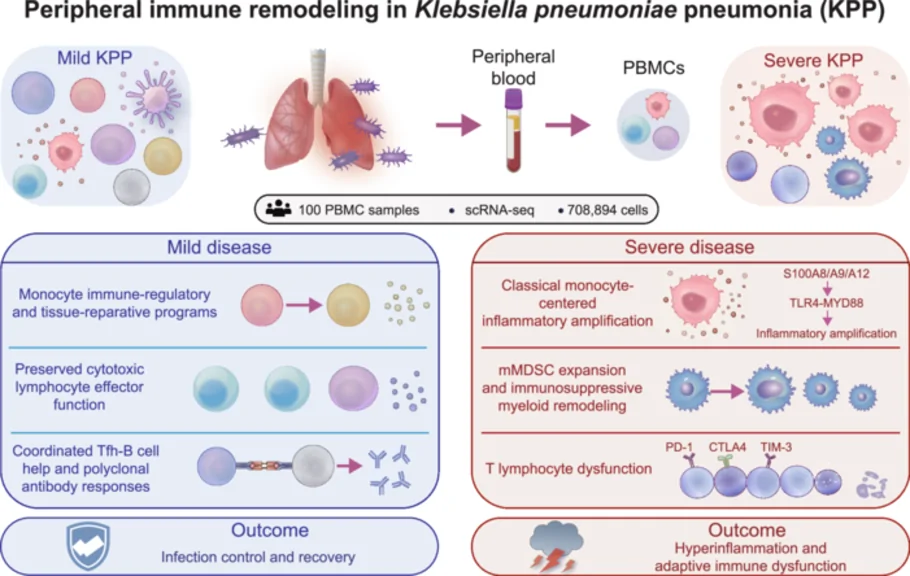

## INTRODUCTION

*Klebsiella pneumoniae* is a common Gram‐negative opportunistic pathogen and an important cause of both hospital‐acquired and community‐acquired pneumonia (CAP) [1]. In China, a multi‐center surveillance study reported *K. pneumoniae* in nearly 7% of CAP cases, ranking it second only to *Streptococcus pneumoniae* [2]. In Asian populations, *K. pneumoniae*‐associated CAP is often severe, with reported mortality rates of 23.8%–27.9%, and is frequently complicated by sepsis and disseminated infection [3, 4]. The increasing prevalence of carbapenem‐resistant and hypervirulent *K. pneumoniae* strains has further narrowed treatment options and increased the clinical burden of this infection [5, 6]. Pathogen‐focused sequencing has improved pathogen detection, antimicrobial‐resistance surveillance, and outbreak tracking [7], but provides limited insight into the host immune states that distinguish mild from severe disease.

Clinical severity in *K. pneumoniae* pneumonia (KPP) is not determined by the pathogen alone. Host immune responses play an equally important role in determining whether infection remains controlled or progresses to severe disease [8, 9]. Work in other Gram‐negative bacterial infections has linked disease progression to concurrent changes in inflammatory, immunoregulatory, and metabolic responses [10]. A properly coordinated immune response is required for bacterial clearance. However, excessive inflammation can damage the lung, disrupt vascular barriers, and worsen respiratory failure [11, 12]. At the same time, severe infection may also impair immune functions that are needed for durable pathogen control, including antigen presentation, T cell coordination, cytotoxic lymphocyte persistence, and antibody responses [13]. This creates a central paradox in severe KPP (SKPP): patients may show strong inflammation while still failing to mount an effective and coordinated immune defense.

Although *K. pneumoniae* infection has been studied from clinical, microbial, and inflammatory perspectives, less is known about how systemic immunity reorganizes as pneumonia becomes severe [14]. Recent single‐cell studies have described immune remodeling in bacterial pneumonia [15], carbapenem‐resistant *K. pneumoniae* (CRKP) pneumonia [16], and *K. pneumoniae* sepsis [17], but a severity‐focused peripheral immune model for KPP remains lacking. In particular, the main cellular source of inflammatory amplification in SKPP and its connection to adaptive immune failure remain unclear. Addressing this gap requires moving beyond bulk cytokines and immune‐cell counts to define the specific cell states and communication programs that separate controlled host defense from immune breakdown [18].

We used single‐cell transcriptomic profiling of peripheral blood mononuclear cells (PBMCs) from 100 individuals, including 54 healthy controls (HCs), 23 patients with mild KPP (MKPP), and 23 patients with SKPP, together with plasma protein measurements and exploratory flow cytometric assessment. We asked whether SKPP represents a distinct immune state rather than a more intense version of mild infection. Our analysis identifies a severity‐linked immune imbalance characterized by *S100A8/A9/A12*‐high inflammatory monocytes, impaired antigen‐presenting programs, suppressive myeloid remodeling, and progressive failure of T cell and B cell coordination. These findings provide a cellular framework for understanding why SKPP combines hyperinflammation with ineffective adaptive immunity, and highlight monocyte‐centered inflammatory amplification as a candidate pathway for future mechanistic and translational investigation.

## RESULTS

### A severity‐focused single‐cell atlas reveals divergent immune states in KPP

To understand how peripheral immunity is reshaped as KPP becomes severe, we profiled PBMCs across the disease spectrum. The final cohort included 100 individuals, comprising 54 HCs, 23 patients with MKPP, and 23 patients with SKPP (Figure 1A, Figure S1, and Table S1). After quality control, 708,894 high‐quality cells were retained for analysis, including 260,121 (36.69%) cells from HC, 220,846 (31.15%) from MKPP, and 227,927 (32.15%) from SKPP. On average, each cell yielded 5004 unique molecular identifiers (UMIs) and 1752 detected genes, supporting high‐confidence downstream immune‐state analysis (Figure S2).

**Figure 1:**
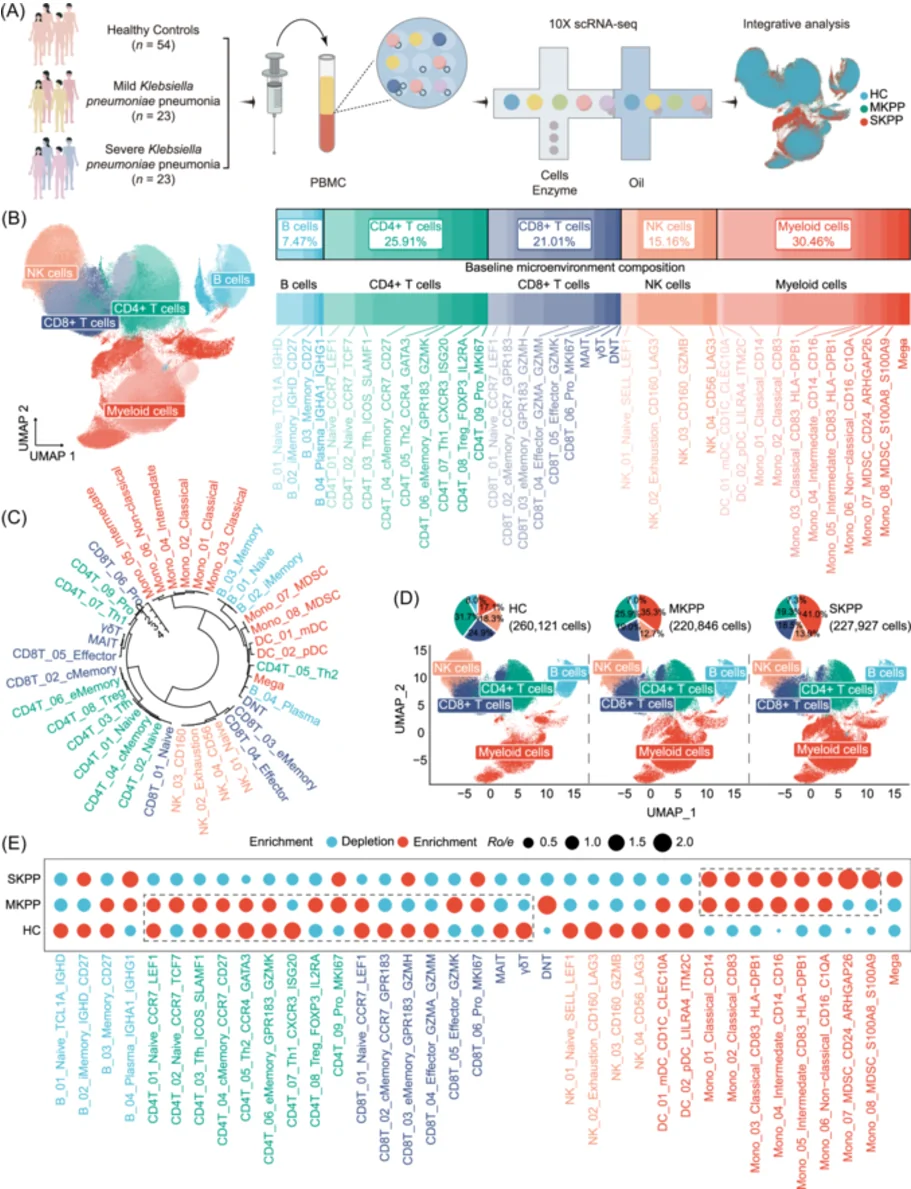
A single‐cell atlas of the peripheral immune response to *Klebsiella pneumoniae* pneumonia. (A) Schematic overview of the study design. PBMCs were profiled from 100 individuals, including 54 healthy controls and 46 patients with KPP, comprising 23 MKPP and 23 SKPP cases. Graphical elements in this schematic were created using Figdraw (https://www.figdraw.com) under publication license No. OOWOY00ddc. (B) UMAP visualization of 708,894 high‐quality PBMCs from all participants, colored by 37 annotated immune cell subtypes. (C) Hierarchical clustering of the 37 immune cell subtypes based on their global transcriptional profiles, showing lineage‐level organization. (D) UMAP projections of all cells split and colored by disease condition, showing the distribution of cells from HC, MKPP, and SKPP groups. (E) Dot plot illustrating compositional changes of the 37 cell subtypes across disease conditions. *R*_*O/E*_ values were calculated as observed cell counts divided by expected cell counts. Dot color indicates depletion or enrichment relative to the expected frequency, and dot size corresponds to the magnitude of the *R*_*O/E*_ value. Cell subtypes are grouped by major lineage. HC, healthy controls; KPP, *K. pneumoniae* pneumonia; MKPP, mild KPP; PBMCs, peripheral blood mononuclear cells; *R*_*O/E*_, ratio of observed to expected; SKPP, severe KPP; UMAP, Uniform Manifold Approximation and Projection.

After batch correction and integration, the single‐cell profiles resolved the major immune compartments in peripheral blood (Figure S3A). Unsupervised clustering identified 11 major cell populations, including B cells, plasma cells, CD4^+^ T cells, CD8^+^ T cells, mucosal‐associated invariant T (MAIT) cells, γδ T cells, natural killer (NK) cells, dendritic cells (DCs), double‐negative T (DNT) cells, monocytes, and megakaryocytes (Figures S3B,C). These lineages showed clear transcriptional separation in Uniform Manifold Approximation and Projection (UMAP) space, with T/NK, B cell, and myeloid compartments occupying distinct regions (Figure 1B and Figure S3B). Further subclustering resolved 37 immune cell subtypes, defined by canonical markers and differentially expressed genes (Figure 1B and Table S2), and hierarchical clustering confirmed their lineage relationships and functional relatedness (Figure 1C).

A major feature of SKPP was a broad reshaping of circulating immunity, with lymphoid populations giving way to an expanded monocyte compartment. Across the disease spectrum, the immune landscape shifted progressively, culminating in a pronounced lymphopenia–monocytosis pattern in SKPP (Figure 1D and Figure S3D). *R*_*O/E*_ (ratio of observed to expected) confirmed this severity‐linked redistribution, identifying lymphopenia and monocytosis as defining features of severe infection (Figure S3E). This transcriptomic pattern was mirrored by clinical peripheral blood cell counts, which showed the same lymphocyte–monocyte imbalance in SKPP (Figure S1C and Table S1). Extending the *R*_*O/E*_ analysis to immune subtypes revealed broad depletion of T cell subsets in SKPP, whereas plasma cells and multiple monocyte subsets, including classical and monocytic myeloid‐derived suppressor cell (mMDSC) populations, were preferentially enriched (Figure 1E). These results indicate that SKPP is not simply a stronger version of mild infection; rather, it represents a systemic immune shift from lymphocyte‐rich host defense toward monocyte‐dominant inflammation.

### *S100A8/A9/A12*‐high monocytes form the inflammatory core of severe KPP

The monocyte‐dominant shift in SKPP raised a central question: which immune cells supply the principal inflammatory signal in severe disease? To address this, we scored inflammatory and cytokine programs across immune subsets (Table S3) [19]. These programs were broadly activated in KPP and reached their highest levels in SKPP, indicating a transcriptomic state resembling cytokine storm in severe disease (Figure 2A and Figure S4A). This inflammatory signal was consistent with clinical evidence of systemic inflammation, as C‐reactive protein (CRP) levels were significantly higher in SKPP patients (Figure S1C).

**Figure 2:**
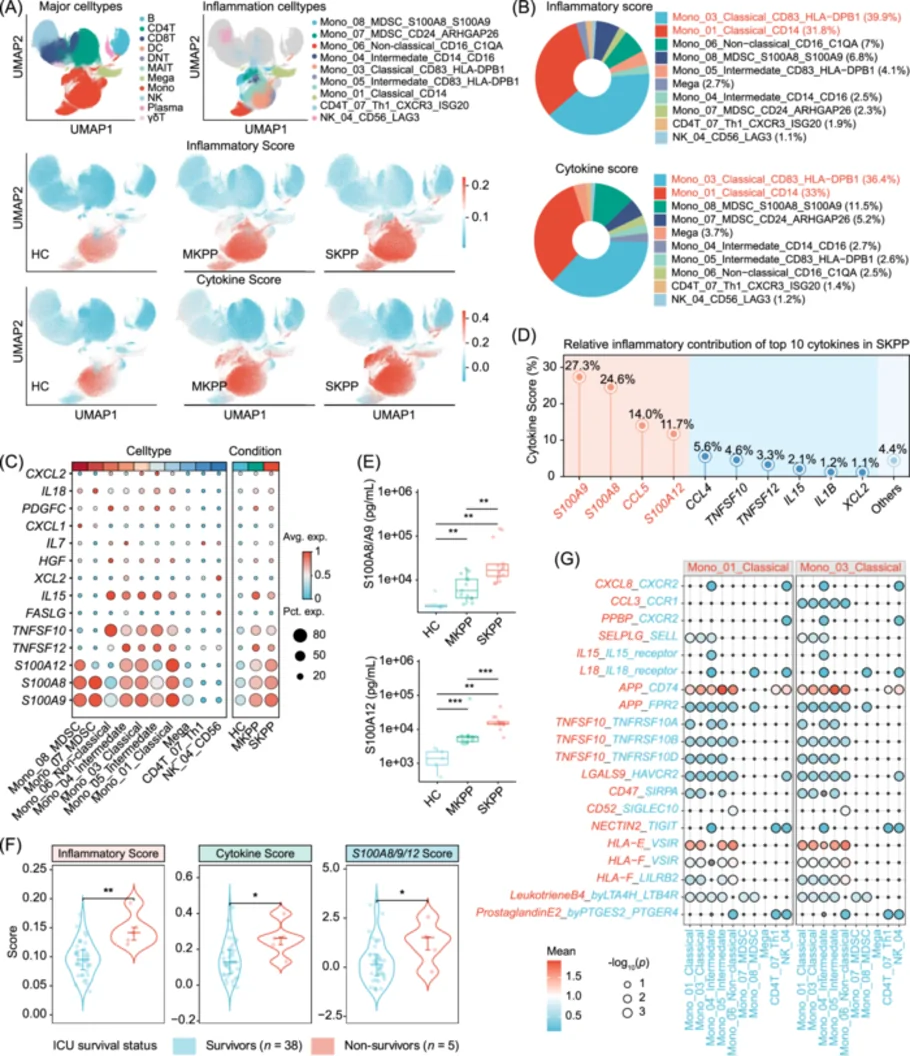
*S100A8/A9/A12*‐high classical monocytes dominate the inflammatory signature of severe *Klebsiella pneumoniae* pneumonia. (A) UMAP visualizations showing major immune cell lineages (top left), identified hyperinflammatory immune cell subtypes (top right), and per‐cell inflammatory and cytokine scores across PBMCs (middle and bottom). The color scale represents score intensity. (B) Pie charts showing the relative contribution of each of the 10 hyperinflammatory immune cell subtypes to the total inflammatory score (top) and cytokine score (bottom) calculated from all cells in SKPP cases. (C) Dot plot showing the expression of representative cytokine and inflammatory genes across hyperinflammatory subtypes and disease conditions. Dot size represents the fraction of cells expressing each gene, and color intensity indicates the average expression level. (D) Lollipop plot ranking the top 10 cytokine genes by their contribution to the total cytokine score in SKPP cases. (E) Plasma concentrations of S100A8/A9 and S100A12 measured by ELISA in HC (*n* = 5), MKPP (*n* = 13), and SKPP (*n* = 14). (F) Violin plots of patient‐level inflammatory, cytokine, and *S100A8/A9/A12* scores in ICU survivors (*n* = 38) and non‐survivors (*n* = 5). Each point represents one patient; central points and error bars indicate medians and IQRs. Groups were compared using two‐sided Wilcoxon rank‐sum tests with Bonferroni correction across the three score comparisons. (G) Dot plot showing CellPhoneDB‐predicted ligand–receptor associations among hyperinflammatory subtypes in pooled SKPP cells. Dot size represents −log_10_(*p* value), and color intensity indicates the mean expression level of the corresponding ligand–receptor pair. Predictions were based on 1000 permutations and a 10% expression threshold. For box plots, the center line represents the median, box limits indicate the IQR, and whiskers extend to 1.5 × IQR. Ligand–receptor interactions were inferred using CellPhoneDB permutation analysis, with significant interactions defined as *p* < 0.05. Statistical significance was assessed using the Kruskal–Wallis test on sample‐level pseudobulk means, with Bonferroni correction for multiple comparisons. ns, *p* > 0.05; *p < 0.05; **p < 0.01; ***p < 0.001. ELISA, enzyme‐linked immunosorbent assay; HC, healthy controls; ICU, intensive care unit; IQR, interquartile range; MKPP, mild *K. pneumoniae* pneumonia; PBMCs, peripheral blood mononuclear cells; SKPP, severe *K. pneumoniae* pneumonia; UMAP, Uniform Manifold Approximation and Projection.

Inflammatory activation in SKPP was highly compartmentalized rather than broadly distributed across immune lineages. Our analysis identified 22 immune cell subtypes, comprising 10 myeloid, 8 T cell, and 4 NK cell subsets, that displayed a distinct pro‐inflammatory phenotype (Figure S4B). Among these, 10 subtypes exhibited particularly potent inflammatory signatures: eight myeloid subsets (including classical, intermediate, and non‐classical monocytes, mMDSC‐like subsets, as well as megakaryocytes), one Th1 subset (CD4T_07_Th1), and one NK subset (NK_04) (Figure 2A and Figure S5). These subtypes showed significantly elevated cytokine and inflammation scores in SKPP, supporting the presence of a restricted set of hyperinflammatory immune states in severe disease. Further dissection revealed that two classical monocyte subsets (Mono_03 and Mono_01) accounted for nearly 70% of the cumulative inflammatory and cytokine scores in SKPP, identifying classical monocytes as the predominant cellular contributors to peripheral inflammatory activation in severe disease (Figure 2B).

This monocyte‐centered pattern is consistent with previous single‐cell studies of bacterial pneumonia, in which classical monocytes were identified as major inflammatory contributors [15]. In SKPP, these inflammatory monocytes were marked by a focused alarmin program, with high expression of *S100A8*/*A9*/*A12* together with other inflammatory mediators, including *TNFSF10/12/13*, *IL1RN*, and *IL15* (Figure 2C and Figure S6A). Among these mediators, *S100A8/A9/A12* showed the clearest disease‐associated pattern, accounting for approximately 63.6% of the cytokine score in SKPP and being predominantly expressed by classical monocytes (Figure 2D and Figure S6B). Their transcriptional elevation was matched by increased plasma concentrations in severe patients, supporting the systemic relevance of this monocyte‐derived alarmin signal (Figure 2E and Figure S6C).

The clinical association of this inflammatory program further supported its relevance to severe disease. *S100A8/A9/A12*, inflammation, and cytokine scores were higher in intensive care unit (ICU) nonsurvivors than in survivors, and these scores correlated positively with plasma procalcitonin (PCT) and CRP (Figure 2F and Figure S6D). Given that *S100* alarmins can signal through *TLR4* and recruit *MYD88* to activate downstream inflammatory pathways [20], we examined this axis in hyperinflammatory monocytes. *TLR4* and *MYD88* were also upregulated in these subsets, and their concurrent expression with *S100A8/A9/A12* was consistent with potential activation of the *S100–TLR4–MYD88* axis in SKPP (Figure S6E).

To delineate intercellular crosstalk underlying this hyperinflammatory state, we mapped ligand–receptor interactions among SKPP monocyte subsets. Interaction count analysis revealed a clear hierarchy of connectivity, with classical monocytes (Mono_01 and Mono_03) emerging as major connectivity hubs, whereas mMDSC populations displayed more restricted interactions (Figure S6F). The predicted network was dominated by chemokine interactions (e.g., *CXCL8–CXCR2* and *CCL3–CCR1*) and TNF‐related signaling (e.g., *TNFSF10–TNFRSF10A/B*), alongside lipid mediator pathways (*LTB4–LTB4R* and *PGE2–PTGER4*) and immunoregulatory axes (e.g., *LGALS9–HAVCR2* and *CD47–SIRPA*) (Figure 2G). These predicted interactions involved pathways associated with leukocyte recruitment, monocyte activation, and inhibitory feedback. Thus, SKPP is characterized by a *S100A8/A9/A12*‐high classical monocyte program that forms the inflammatory core of the circulating immune response.

### Severe KPP redirects CD4^+^ T cells into a poorly coordinated activated state

CD4^+^ T cells sit at the center of antimicrobial immunity, bridging inflammatory sensing, the provision of T cell help, and immune regulation [21]. In KPP, this coordinating function appeared to erode progressively as infection advanced toward severe disease. Sub‐clustering resolved nine CD4^+^ T cell subtypes, including two naïve populations (CD4T_01_Naive and CD4T_02_Naive), regulatory (CD4T_08_Treg), follicular helper (CD4T_03_Tfh), Th2 (CD4T_05_Th2), central memory (CD4T_04_cMemory), effector memory (CD4T_06_eMemory), proliferative (CD4T_09_Pro), and Th1 (CD4T_07_Th1) subsets (Figure 3A and Figure S7A). By *R*_*O/E*_ analysis, several helper and regulatory subsets were preferentially enriched in MKPP, whereas the proliferative subset expanded across the KPP spectrum and peaked in severe disease (Figure 1E). CD4^+^ T cell remodeling in KPP therefore reflected not a straightforward loss of helper cells but a shift in composition toward activated and proliferative states.

**Figure 3:**
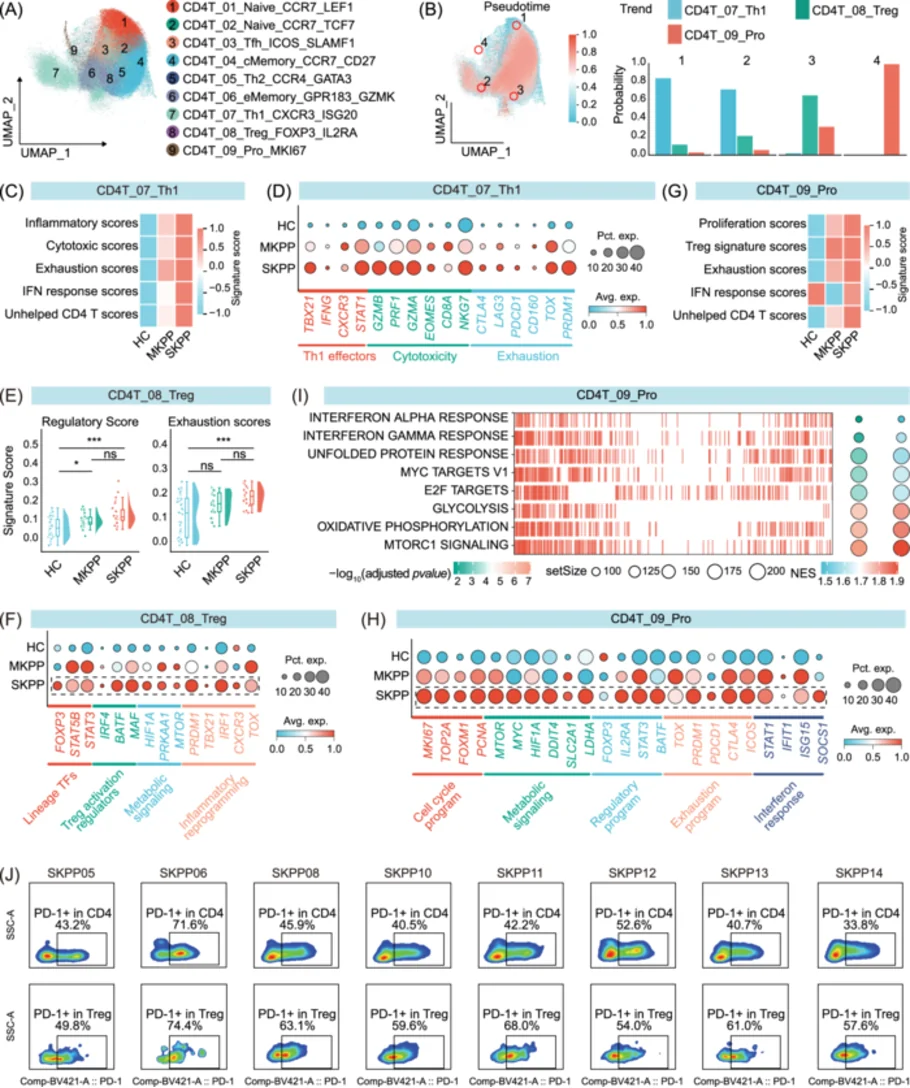
Severe *Klebsiella pneumoniae* pneumonia reshapes CD4^+^ T cells toward a poorly coordinated activated state. (A) UMAP visualization of CD4^+^ T cell subsets (left) and annotation of nine transcriptionally defined subtypes (right). (B) Palantir‐based pseudotime analysis of CD4^+^ T cells. The left panel shows pseudotime distribution along the inferred differentiation landscape, and the right panel shows lineage probability trends toward CD4T_07_Th1, CD4T_08_Treg, and CD4T_09_Pro terminal states. (C) Heatmap showing functional module scores of CD4T_07_Th1 cells across disease conditions. (D) Dot plot showing the expression of representative CD4T_07_Th1 effector‐, cytotoxic‐, and exhaustion‐associated genes across disease conditions. Dot size represents the fraction of cells expressing each gene, and color intensity indicates the average expression level. (E) Raincloud plots showing functional module scores of CD4T_08_Treg cells across disease conditions. (F) Dot plot showing the expression of lineage‐defining transcription factors, activation regulators, metabolic signaling genes, and inflammatory reprogramming markers in CD4T_08_Treg cells across disease conditions. (G) Heatmap showing functional profiling of CD4T_09_Pro cells across disease conditions. (H) Dot plot showing expression of representative genes involved in cell cycle, metabolic signaling, regulatory programs, exhaustion and interferon response in CD4T_09_Pro cells across disease conditions. (I) GSEA of CD4T_09_Pro cells, showing severity‐associated pathway enrichment. (J) Representative flow cytometry plots showing exhaustion‐associated PD‐1 expression in CD4^+^ T cells and Tregs from eight SKPP patients. Numbers indicate the percentage of PD‐1^+^ cells within the gated CD4^+^ T cell or Treg population. Statistical significance was assessed using the Kruskal–Wallis test on sample‐level pseudobulk means, with Bonferroni correction for multiple comparisons. ns, *p* > 0.05; *p < 0.05; ***p < 0.001. GSEA, Gene Set Enrichment Analysis; SKPP, severe *K. pneumoniae* pneumonia; UMAP, Uniform Manifold Approximation and Projection.

Trajectory analysis indicated that these altered states were interconnected rather than arising independently. Partition‐based graph abstraction (PAGA) positioned memory cells at the hub of the CD4^+^ T cell landscape, connecting them to Th1, Th2, and Treg branches, with proliferative cells most closely tied to the Treg trajectory (Figure S7B). Palantir analysis resolved three dominant fate trajectories, directed toward Th1, Treg, and proliferative states (Figure 3B). Functional scoring was consistent with this structure: Th1 cells retained inflammatory and cytotoxic features, Tregs showed regulatory activation, and CD4T_09_Pro co‐expressed proliferation, Treg‐associated, exhaustion, interferon‐response, and T cell receptor (TCR) signaling programs (Figures S7C,D and Table S4). These patterns describe a redirection of CD4^+^ T cells in KPP, away from coordinated helper differentiation and toward mixed activation–regulatory programs.

This breakdown in coordination was most apparent in the Th1 compartment. Across the HC‐to‐SKPP gradient, Th1 cells acquired progressively stronger inflammatory, interferon‐response, and cytotoxic signatures, alongside rising “unhelped CD4 T” scores (Figure 3C and Figure S7C). Th1 cells in SKPP preserved their lineage identity yet took on more pronounced cytotoxic and inhibitory features, with upregulation of *GZMB*, *PRF1*, *PDCD1*, and *CTLA4* and heightened activity of the *STAT1*, *IRF1*, and *TBX21* regulons (Figure 3D and Figure S7E). Because CD4^+^ T cells can adopt cytotoxic programs under inflammatory conditions [22, 23], this profile is consistent with a sustained inflammatory‐cytotoxic Th1 state in SKPP, coupled with inhibitory‐receptor upregulation and weakened helper coordination.

The Treg compartment was likewise reshaped in SKPP. These cells became more abundant and carried higher regulatory scores in severe disease (Figure 3E). They retained a canonical regulatory identity, with elevated *FOXP3* and *IL2RA*, while simultaneously adopting an activated phenotype marked by co‐stimulatory and activation‐related molecules such as *ICOS* and *TNFRSF18* (Figure 3F and Figure S7F). Importantly, these Tregs did not correspond to a purely resting regulatory phenotype. Instead, they additionally acquired exhaustion‐associated features, signs of inflammatory reprogramming, and enhanced metabolic signaling (Figure 3F). Flow cytometry corroborated these exhaustion‐associated features in Tregs from SKPP patients at the protein level (Figure 3J and Figure S8). This indicates that Tregs in SKPP are not merely activated but transcriptionally remodeled by the inflammatory milieu, holding onto their regulatory identity while layering on stress‐ and exhaustion‐related programs.

Of all the CD4^+^ T cell states, the proliferative subset (CD4T_09_Pro) tracked most closely with disease progression. It expanded in both MKPP and SKPP and sat adjacent to the Treg branch, yet amounted to more than a simple cycling population (Figures S7B,D; S7G). Instead, CD4T_09_Pro combined proliferation, Treg‐associated, exhaustion, interferon‐response, and TCR‐signaling programs with elevated “unhelped CD4 T” scores (Figure 3G and Figure S7C). In severe disease, these cells upregulated cell‐cycle genes (e.g., *MKI67*, *TOP2A*), metabolic regulators (e.g., *MTOR*, *MYC*), regulatory molecules (e.g., *IL2RA*, *STAT3*), exhaustion‐associated markers (e.g., *PDCD1*, *CTLA4*) and interferon‐response genes (e.g., *STAT1*, *ISG15*) (Figure 3H). Pathway analysis revealed further amplification of *mTOR*, *MYC*, *E2F*, interferon‐response, metabolic, and protein‐stress programs in SKPP relative to MKPP (Figure 3I, Figure S7H, and Tables S5–S10). Mfuzz analysis reinforced this severity‐linked proliferative program, isolating a gene module (Cluster 4) whose expression rose from HC to SKPP and was enriched for DNA replication, mitotic division, and DNA repair (Figures S7I,J and Table S11). Taken together, these analyses position CD4T_09_Pro at the convergence of proliferation, inflammatory activation, regulatory programming, exhaustion‐associated signaling, and impaired helper function.

CD4^+^ T cell dysfunction in SKPP is therefore poorly captured by the notions of simple depletion or uniform exhaustion. Th1 and Treg subsets underwent lineage‐specific remodeling, whereas CD4T_09_Pro represented a convergence state that combined proliferation, inflammatory activation, regulatory features, exhaustion‐associated signals, and impaired helper programming. Rather than sustaining coordinated helper immunity, severe disease pushed CD4^+^ T cells into a hyperactivated but poorly coordinated state, in which the proliferative subset marked the collapse of effective helper function.

### Severe KPP is associated with a cytotoxic lymphocyte state bearing exhaustion‐associated features

CD8^+^ T cells and innate‐like cytotoxic lymphocytes are central effectors of antimicrobial defense [24], but their protective value rests on sustained cytotoxic activity that does not tip prematurely into dysfunction. We resolved eight CD8‐lineage populations—six conventional CD8^+^ T cell subsets and two innate‐like subtypes (MAIT and γδ T cells)—both of which expressed *CD8A/CD8B* and were therefore analyzed alongside the CD8 compartment (Figure 4A, Figure S9A, and Table S2). The conventional subsets spanned naïve (CD8T_01_Naive), central memory (CD8T_02_cMemory), effector memory (CD8T_03_eMemory), two effector states (CD8T_04_Effector and CD8T_05_Effector), and proliferating (CD8T_06_Pro) cells (Figure 4A and Figure S9A). By *R*_*O/E*_ analysis, effector and innate‐like populations sat preferentially in HCs, whereas the proliferative CD8^+^ T cell subset expanded in KPP and was most prominent in severe disease (Figure 1E). Together, these shifts pointed to a disturbance of the cytotoxic lymphocyte pool as infection progressed.

**Figure 4:**
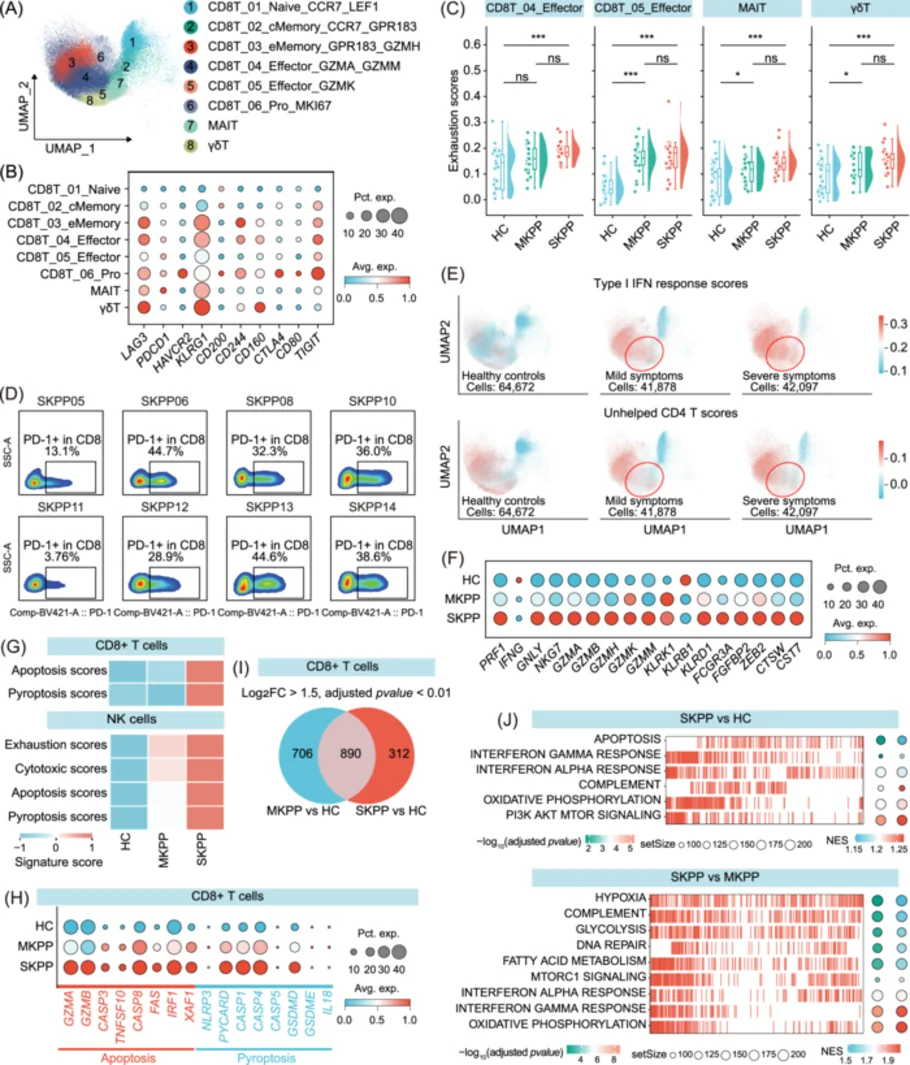
Severe *Klebsiella pneumoniae* pneumonia is associated with CD8‐lineage lymphocyte states bearing cytotoxicity‐ and exhaustion‐related signatures. (A) UMAP visualization of CD8^+^ T cell subsets (left) and annotation of eight transcriptionally defined subtypes (right). (B) Dot plot showing the expression of selected exhaustion‐associated markers across CD8‐lineage lymphocyte subtypes. Dot size represents the fraction of cells expressing each gene, and color intensity indicates the average expression level. (C) Raincloud plots showing exhaustion scores in CD8T_04_Effector, CD8T_05_Effector, MAIT, and γδ T cells across disease conditions. (D) Representative flow cytometry plots showing exhaustion‐associated PD‐1 expression in CD8^+^ T cells from eight SKPP patients. Numbers indicate the percentage of PD‐1^+^ cells within the gated CD8^+^ T cell population. (E) UMAP visualizations showing type I interferon response scores (top) and unhelped CD4^+^ T cell signature scores (bottom) across disease conditions. Red circles highlight regions with increased signature activity in MKPP and SKPP. (F) Dot plot showing the expression of representative cytotoxicity‐associated genes across disease conditions. (G) Heatmap showing apoptosis‐ and pyroptosis‐related scores in CD8^+^ T cells (top), and exhaustion‐, cytotoxicity‐, apoptosis‐, and pyroptosis‐related scores in NK cells (bottom) across disease conditions. (H) Dot plot showing the expression of selected apoptosis‐ and pyroptosis‐related genes in CD8^+^ T cells across disease conditions. (I) Venn diagram illustrating the overlap of upregulated DEGs in CD8^+^ T cells from MKPP versus HC and SKPP versus HC comparisons. (J) GSEA plots showing enrichment of Hallmark pathways in CD8^+^ T cells. Top panels compare SKPP versus HC, and bottom panels compare SKPP versus MKPP. Enriched pathways are shown as barcode plots, with dot size indicating gene‐set size, dot color indicating NES, and the heatmap‐like bar indicating −log_10_(adjusted *p* value). Statistical significance was assessed using the Kruskal–Wallis test on sample‐level pseudobulk means, with Bonferroni correction for multiple comparisons. ns, *p* > 0.05; *p < 0.05; ***p < 0.001. DEGs, differentially expressed genes; GSEA, Gene Set Enrichment Analysis; HC, healthy controls; MKPP, mild *K. pneumoniae* pneumonia; NES, normalized enrichment score; SKPP, severe *K. pneumoniae* pneumonia; UMAP, Uniform Manifold Approximation and Projection.

Exhaustion‐associated remodeling was the defining feature of this compartment in SKPP. Effector CD8^+^ T cell subsets and innate‐like cytotoxic populations carried the highest exhaustion scores, and these rose further in SKPP relative to HC and MKPP (Figure 4B,C and Figure S9B). Within the exhaustion‐associated cells, SKPP patients showed elevated inhibitory receptors (e.g., *PDCD1*, *LAG3*, and *HAVCR2* (TIM‐3)) alongside increased *PRDM1* (Blimp‐1), a transcription factor tied to inhibitory‐receptor expression and reduced T cell polyfunctionality (Figure S9C) [15, 25, 26, 27]. Because such inhibitory programs constrain T cell proliferation and cytokine output, the profile fits an exhaustion‐associated state rather than transient activation. Flow cytometry detected PD‐1‐positive CD8^+^ T cells in eight SKPP patients (Figure 4D and Figure S10), providing orthogonal evidence for the presence of this exhaustion‐associated marker at the protein level. Together with the transcriptomic findings, these results support exhaustion‐associated remodeling of the cytotoxic lymphocyte compartment in SKPP.

This exhausted compartment was far from inert. It instead displayed a paradoxical profile in which markers of dysfunction sat alongside preserved cytotoxic potential. CD8^+^ T cells with exhaustion‐associated features were enriched for type I IFN‐related genes, in keeping with the persistent inflammatory stimulation of severe disease (Figure 4E). The same cells also carried higher “unhelped” CD8^+^ T cell scores, indicating insufficient CD4^+^ T cell support within the exhausted pool (Figure 4E). Taken together, these features were consistent with persistent inflammatory pressure and reduced helper‐associated support in SKPP CD8^+^ T cells. Yet despite this unfavorable setting, four exhaustion‐associated subsets held on to strong cytotoxic gene signatures, and core effector molecules (e.g., *CST7*, *GNLY*, *PRF1*, *GZMA/GZMB*, *NKG7*) were further upregulated in SKPP patients (Figure 4F and Figures S9B,D). Such retention of killing capacity despite blunted proliferation and cytokine production echoes earlier reports on exhausted CD8^+^ T cells [28]. SKPP thus carries a cytotoxic‐exhausted state in which the machinery for killing persists but is poorly coupled to durable protective immunity.

The persistence of these cytotoxic programs in SKPP came at a cost. Sustained inflammatory stimulation renders cytotoxic lymphocytes vulnerable to activation‐induced cell death, and that vulnerability surfaced in severe disease. CD8^+^ T cells co‐expressing exhaustion and cytotoxicity programs showed elevated apoptosis‐related scores, peaking in SKPP patients (Figure 4G and Figure S9E). Accompanying this was the coordinated upregulation of genes spanning granzyme/perforin‐, *TNF*‐, *XAF1*‐, and *FAS*‐associated apoptotic pathways, including *GZMA/B*, *TNFRSF10*, *IRF1*, *FAS*, *CASP3/8*, and *XAF1* (Figure S9F). The same stress signature extended to selected inflammasome‐ and gasdermin‐associated genes in SKPP CD8^+^ T cells—*NLRP3*, *PYCARD*, *CASP1*, *GSDMD*, *GSDME*, and *IL18* (Figures 4G,H). Rather than marking overt pyroptosis, these features describe an inflammatory cell‐death–related stress state within the cytotoxic compartment. NK cells mirrored this pattern, combining higher exhaustion scores with apoptosis‐ and pyroptosis‐related signatures (Figure 4G). In short, SKPP does not simply amplify cytotoxic immunity; it gives rise to a fragile cytotoxic state in which effector activation coexists with exhaustion, apoptotic priming, and inflammatory cell‐death stress.

The transcriptional backdrop to this fragile state pointed to a broader interferon–metabolic stress program. Among exhaustion‐prone CD8‐lineage subsets, cells from MKPP and SKPP samples showed 1596 and 1202 upregulated genes, respectively, relative to cells from HCs, with 890 shared between the two disease groups (Figure 4I, Tables S12 and S13). MKPP carried a narrower enrichment profile centered on protein secretion and PI3K–AKT–mTOR signaling, in line with moderate adaptive activation (Figure S9G and Table S14). SKPP, by contrast, engaged a wider response spanning interferon‐α/γ responses, complement, oxidative phosphorylation, MYC targets, and PI3K–AKT–mTOR signaling (Figure 4J and Table S15). A direct SKPP‐versus‐MKPP comparison singled out oxidative phosphorylation, mTORC1 signaling, fatty acid metabolism, glycolysis, hypoxia, and interferon responses as severity‐associated programs (Figure 4J and Table S16). SKPP therefore does more than raise the level of cytotoxic‐cell activation—it overlays an interferon–metabolic stress program on exhaustion‐prone CD8‐lineage cells.

### Severe KPP decouples plasma cell expansion from coordinated humoral immunity

In KPP, humoral immunity shifted toward plasma cell accumulation as disease worsened, yet this expansion did not, by itself, signal a coordinated antibody response. Using canonical marker expression, we resolved four transcriptionally distinct B cell subtypes: naïve (B_01_Naive), intermediate memory (B_02_iMemory), memory (B_03_Memory), and plasma cells (B_04_Plasma) (Figure 5A, Figure S11A, and Table S2). Both *R*_*O/E*_ analysis and cell‐composition profiling traced a progressive contraction of naïve B cells alongside an expansion of plasma cells across HC, MKPP, and SKPP (Figures 1E and 5B). This compositional pattern was consistent with increased representation of terminally differentiated plasma cells, most prominently in SKPP.

**Figure 5:**
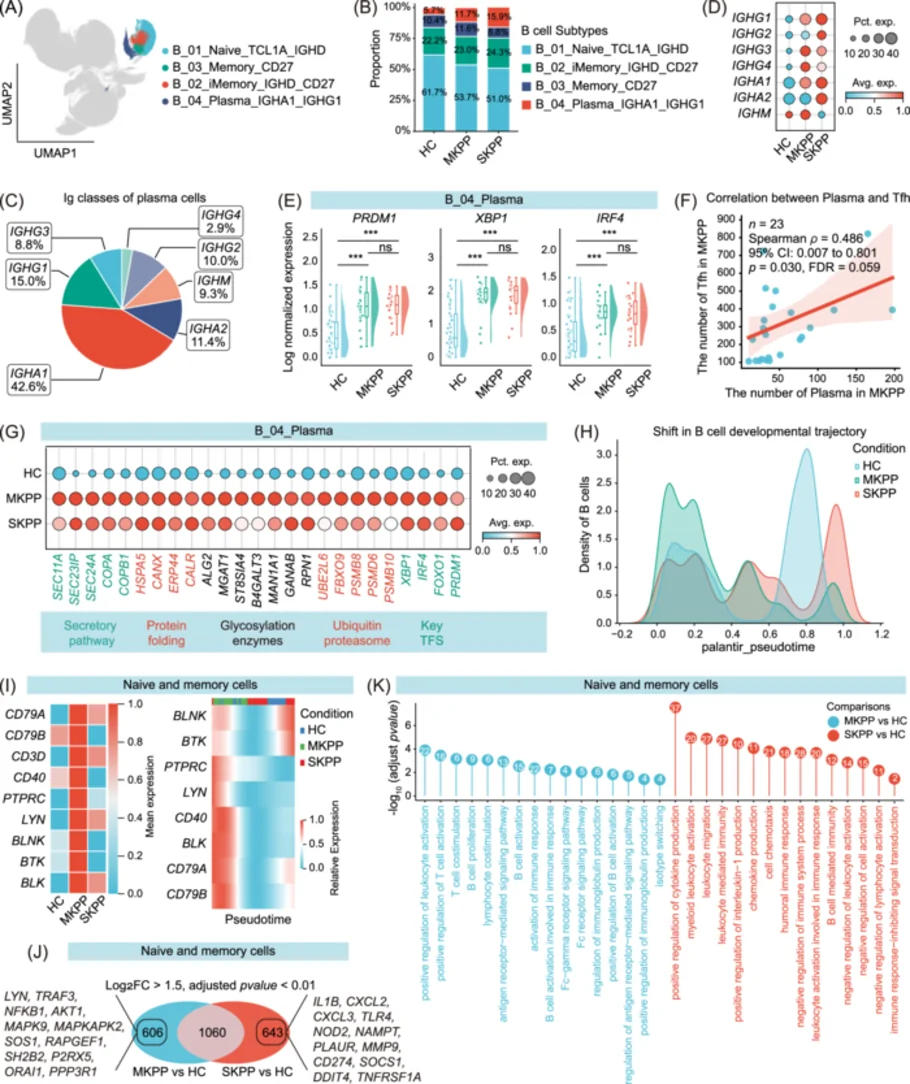
Severe *Klebsiella pneumoniae* pneumonia decouples plasma cell expansion from coordinated humoral immunity. (A) UMAP visualization of B cell subsets (left) and annotation of four transcriptionally defined subtypes (right). (B) Stacked bar plot showing the relative composition of B cell subtypes across disease conditions. (C) Pie chart showing the distribution of immunoglobulin heavy chain classes in plasma cells. (D) Dot plot showing the expression of selected immunoglobulin heavy chain genes in plasma cells across disease conditions. Dot size represents the fraction of cells expressing each gene, and color intensity indicates the average expression level. (E) Raincloud plots showing the expression of plasma cell transcription factors *PRDM1*, *XBP1*, and *IRF4* in B_04_Plasma cells across disease conditions. (F) Patient‐level correlation between plasma cell and Tfh cell counts in MKPP. Each point represents one patient. Spearman's *ρ*, bootstrap 95% CI, nominal *p* value, and BH‐FDR‐adjusted *p* value are shown. FDR correction was performed across the prespecified MKPP and SKPP correlations. (G) Dot plot showing the expression of selected secretory pathway‐, protein folding‐, glycosylation‐, ubiquitin–proteasome‐, and transcription factor‐related genes in B_04_Plasma cells across disease conditions. (H) Density plot showing the distribution of B cells along the inferred developmental pseudotime across disease conditions. (I) Heatmap showing the expression of selected BCR signaling‐related genes in naïve/memory B cells across disease conditions (left), and heatmap showing pseudotime‐dependent expression dynamics of these genes along the B cell trajectory (right). (J) Venn diagram showing overlap of differentially expressed genes in naïve/memory B cells between MKPP versus HC and SKPP versus HC comparisons. Representative MKPP‐specific and SKPP‐specific genes are shown beside the diagram. (K) GO enrichment analysis of MKPP‐specific and SKPP‐specific genes in naïve/memory B cells. Blue terms represent pathways enriched among MKPP‐upregulated genes, and red terms represent pathways enriched among SKPP‐upregulated genes. Numbers above bars indicate the number of genes contributing to each GO term. Statistical significance was assessed using the Kruskal–Wallis test on sample‐level pseudobulk means, with Bonferroni correction for multiple comparisons. ns, *p* > 0.05; ***p < 0.001. BCR, B cell receptor; GO, Gene Ontology; HC, healthy controls; MKPP, mild *K. pneumoniae* pneumonia; SKPP, severe *K. pneumoniae* pneumonia; UMAP, Uniform Manifold Approximation and Projection.

Plasma cells carried a transcriptional program associated with antibody secretion that spanned all major immunoglobulin heavy‐chain classes, including *IGHA1*, *IGHA2*, *IGHG1*, *IGHG2*, *IGHG3*, *IGHG4*, and *IGHM* (Figure 5C). *IGHA1* was the dominant immunoglobulin heavy‐chain transcript (Figure 5C), and both *IGHA1* and *IGHA2* rose further in SKPP than in MKPP, pointing to an IgA‐skewed immunoglobulin transcriptional pattern in severe disease that fits the mucosal origin of KPP (Figure 5D) [29]. *IGHG3*, *IGHG4*, and *IGHM*, by contrast, were more prominent in MKPP, in keeping with a broader immunoglobulin heavy‐chain transcript profile in MKPP (Figure 5D). The plasma cell identity factors *PRDM1*, *XBP1*, and *IRF4* were elevated in both MKPP and SKPP relative to HC but were not induced appreciably further in SKPP (Figure 5E). Severe disease was therefore set apart less by a stronger differentiation program than by greater plasma cell accumulation and IgA‐biased immunoglobulin heavy‐chain transcript usage.

Effective humoral immunity rests not only on plasma‐cell expansion but also on coordinated Tfh–B cell communication [30]. In MKPP, circulating Tfh cells were expanded and correlated positively with plasma cell frequency, in line with a helper‐linked plasma cell response during mild disease (Figures 1E and 5F). In SKPP, this correlation weakened and lost statistical significance, indicating a looser coupling between plasma cell accumulation and Tfh support (Figure S11B). The loss of intercellular coordination was mirrored at the transcriptional level: genes and transcription factors governing antibody transport, secretion, folding, and protein stability were expressed more highly in MKPP than in SKPP, consistent with weaker secretory machinery in severe disease (Figure 5G). MKPP thus retained a coordinated, Tfh‐linked antibody‐secreting program, whereas SKPP combined plasma cell expansion with poorer preservation of this secretory machinery.

B cell developmental trajectories sharpened the divide between mild and severe disease. MKPP retained more B cells in early and intermediate pseudotime states, whereas SKPP was skewed toward late states, mirroring the plasma cell accumulation seen in severe disease (Figure 5H and Figure S11C). This terminal shift coincided with reduced antigen‐recognition programs outside the plasma cell compartment. In naïve and memory B cells, core B cell receptor (BCR) signaling genes (*CD79A*, *CD79B*, *CD40*, *LYN*, *BLNK*, *BTK*, and *BLK*) were more highly expressed in MKPP but were expressed at lower levels in SKPP (Figure 5I and Figure S11D). Along pseudotime, these BCR‐related genes fell as cells advanced toward late states, linking the plasma cell skewing of SKPP with reduced expression of antigen‐responsive B cell transcriptional programs (Figure 5I).

This MKPP–SKPP contrast held even after plasma cells were removed from the comparison. Within naïve and memory B cells, the MKPP‐specific genes were dominated by receptor and downstream signaling molecules, including *LYN*, *TRAF3*, *NFKB1*, *AKT1*, *MAPK9*, *MAPKAPK2*, *SOS1*, *RAPGEF1*, *SH2B2*, *ORAI1*, and *PPP3R1* (Figure 5J and Table S17). These genes were enriched for B cell activation, antigen‐receptor–mediated signaling, lymphocyte co‐stimulation, B cell proliferation, immunoglobulin production, and isotype switching, defining a transcriptional program associated with B cell activation in mild disease (Figure 5K and Table S19). The SKPP‐specific genes told a different story. They comprised inflammatory mediators and innate immune sensors (*IL1B*, *CXCL2*, *CXCL3*, *TLR4*, *NOD2*, *NAMPT*, *PLAUR*, and *MMP9*), alongside regulatory and stress‐associated molecules such as *CD274* (PD‐L1), *SOCS1*, *DDIT4*, and *TNFRSF1A* (Figure 5J and Table S18). Their enriched functions spanned cytokine production, leukocyte activation and migration, the humoral immune response, and immune‐inhibitory signaling (Figure 5K and Table S20). In SKPP, naïve and memory B cells showed reduced antigen‐responsive programs together with increased inflammatory, stress‐related, and immune‐inhibitory signatures.

### Myeloid remodeling links inflammatory amplification with antigen‐presentation failure and immune suppression in severe KPP

Myeloid cells coordinate early antibacterial defense, antigen presentation, and inflammatory regulation [31]. Given the monocyte‐dominant shift seen in SKPP, we reanalyzed the myeloid compartment to define how these cells were reorganized across disease severity. The analysis resolved 11 myeloid‐associated populations: eight monocyte subsets, two dendritic cell (DC) subsets, and megakaryocytes (Figure 6A, Figure S12A, and Table S2). The monocyte compartment spanned classical, intermediate, non‐classical, and mMDSC populations, while the DCs separated into myeloid DCs (mDCs) and plasmacytoid DCs (pDCs) (Figure 6A and Figure S12A). *R*_*O/E*_ analysis revealed a marked restructuring of the myeloid landscape in SKPP, with broad enrichment of monocyte subsets and contraction of DC populations (Figure 1E). This pattern pointed to a myeloid state in which inflammatory expansion coincided with weakened antigen‐presenting capacity.

**Figure 6:**
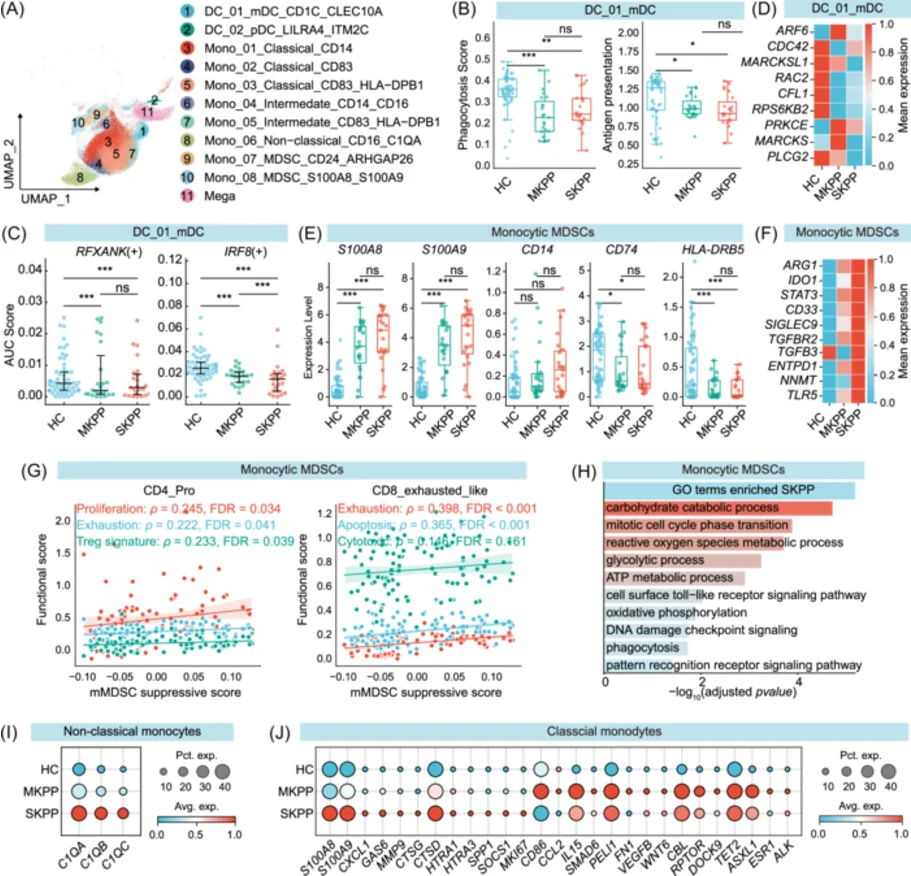
Myeloid remodeling in severe *Klebsiella pneumoniae* pneumonia is characterized by inflammatory amplification, reduced antigen‐presentation programs, and immunoregulatory monocyte states resembling monocytic myeloid‐derived suppressor cells. (A) UMAP visualization of myeloid cell subtypes (left) and annotation of 11 transcriptionally defined subtypes (right). (B) Raincloud plots showing phagocytosis (left) and antigen‐presentation (right) scores in DC_01_mDC cells across disease conditions. (C) AUC scores of *RFXANK*(+) (left) and *IRF8*(+) (right) regulons in DC_01_mDC cells across disease conditions. (D) Heatmap showing the mean expression of selected genes in DC_01_mDC cells. (E) Box plots showing the expression of *S100A8*, *S100A9*, *S100A12*, *CD74*, and *HLA‐DRB5* in mMDSCs across disease conditions. (F) Heatmap showing the mean expression of selected genes in mMDSCs. (G) Scatter plots showing associations between mMDSC immunoregulatory scores and functional states of CD4T_09_Pro cells (left) and CD8_exhausted‐like cytotoxic lymphocytes (right). Each point represents one donor. Spearman's *ρ* and Benjamini–Hochberg FDR‐adjusted *p* values are shown. (H) Bar plot showing representative GO terms enriched among genes upregulated in SKPP‐associated mMDSCs. Bar length indicates −log_10_(adjusted *p* value). (I) Dot plot showing the expression of selected genes in non‐classical monocytes across disease conditions. (J) Dot plot showing the expression of selected genes in classical monocytes across disease conditions. Statistical significance was assessed using the Kruskal–Wallis test on sample‐level pseudobulk means, with Bonferroni correction for multiple comparisons. ns, *p* > 0.05; *p < 0.05; **p < 0.01; ***p < 0.001. AUC, area under the curve; GO, Gene Ontology; mMDSC, monocytic myeloid‐derived suppressor cell; SKPP, severe *K. pneumoniae* pneumonia; UMAP, Uniform Manifold Approximation and Projection.

The loss of antigen‐presenting capacity was clearest in mDCs. As professional antigen‐presenting cells, mDCs bridge innate pathogen sensing and adaptive T cell priming [32], and in KPP this bridge appeared compromised. mDCs from SKPP patients scored lower for phagocytosis and antigen presentation than their counterparts in healthy donors (Figure 6B) and broadly downregulated major histocompatibility complex (MHC) class II genes, most notably *HLA‐DQ* and *HLA‐DR* molecules (Figure S12C). Regulon analysis pointed the same way, showing reduced activity of *RFXANK* and *IRF8*, two regulators tied to MHC class II expression and DC identity (Figure 6C). Genes governing cytoskeletal remodeling and phagocytic signaling, including *CDC42*, *RAC2*, *CFL1*, *RPS6KB2*, and *PLCG2*, were likewise reduced (Figure 6D). Predicted mDC interactions with naïve CD4^+^ T cells through co‐stimulatory pairs such as *ICOSLG–ICOS*, *CD58–CD2*, and *CD86–CD28* nonetheless remained detectable (Figure S12D). SKPP mDCs therefore seemed to retain elements of T cell contact signaling while losing the upstream programs needed for antigen capture, processing, and presentation, consistent with reduced antigen‐processing and antigen‐presentation capacity in severe disease.

A second feature of SKPP myeloid remodeling was the expansion of mMDSC‐like monocytes with immunoregulatory features. Mono_07_MDSC and Mono_08_MDSC were enriched and increased in relative abundance in SKPP (Figure 1E and Figure S12B). These cells displayed a *CD14* ^+^ *HLA‐DR* ^−/low^ phenotype with elevated calprotectin, a profile in keeping with monocytic suppressive myeloid cells (Figure S12E) [33]. Both subsets expressed high *S100A8/A9* and low *HLA‐DR*, supporting their annotation as mMDSC‐like monocyte populations (Figure 6E). In SKPP, they further upregulated immunoregulatory molecules, including *ARG1*, *IDO1*, *STAT3*, *CD33*, *SIGLEC9*, *TGFBR2*, *TGFB3*, and *ENTPD1* (CD39), consistent with an active suppressive program rather than mere myeloid expansion (Figure 6F). Their immunoregulatory scores increased in KPP and correlated with multiple features of T cell dysfunction: proliferation, exhaustion, and Treg‐like programs in CD4T_09_Pro cells; exhaustion and apoptosis in CD8^+^, MAIT, and γδ T cytotoxic lymphocytes; and Th1 exhaustion together with Treg regulatory activity (Figure 6G, Figures S12F,G, and Table S21). Predicted *ENTPD1–ADORA2A* signaling was observed between mMDSCs and CD4T_09_Pro cells, indicating a potential purinergic–adenosinergic interaction associated with these populations (Figure S12H) [34]. The mMDSC program itself shifted with disease severity. MKPP‐associated mMDSCs were enriched for myeloid differentiation, cell adhesion, and leukocyte migration, in line with early myeloid mobilization (Figure S12I and Tables S22 and S24). SKPP‐associated mMDSCs instead showed enrichment for metabolic activation, reactive oxygen species (ROS) stress, cell‐cycle activity, phagocytosis, and TLR or pattern‐recognition‐receptor signaling (Figure 6H and Tables S23 and S25). In SKPP, then, mMDSCs appeared to move past early myeloid mobilization and adopt a metabolically active, inflammatory, and suppressive state coupled to adaptive immune dysfunction.

Non‐classical monocytes added a complement‐linked inflammatory layer to the myeloid response. Mono_06_Non‐classical_CD16_C1QA was the principal circulating source of C1 complement components (Figure S13), and this subset increased its expression of *C1QA/B/C* in SKPP patients (Figure 6I). Complement is indispensable for pathogen clearance, yet its excessive activation can amplify tissue injury and vascular leakage [35]. This C1q‐high non‐classical monocyte program was consistent with increased complement‐associated inflammatory activity in SKPP.

Classical monocytes showed the clearest severity‐dependent split within the myeloid compartment. Although MKPP and SKPP classical monocytes shared 1768 upregulated genes, each disease state retained its own transcriptional program (Figure S12J and Tables S26 and S27). In SKPP, classical monocytes were enriched for inflammatory‐amplification and tissue‐injury genes, including *S100A8/A9*, *CXCL1*, *MMP9*, and *CTSD*, as well as cell‐cycle genes such as *CDK1* and *MKI67* (Figure 6J and Figure S12J). These genes implicate alarmin signaling, neutrophil recruitment, extracellular‐matrix remodeling, and proliferative activation, features consistent with an immunopathogenic monocyte state [36, 37, 38]. The aberrant expression of cell‐cycle genes (*CDK1*, *MKI67*) in particular suggests dysregulated proliferation in the periphery. In sharp contrast, classical monocytes from MKPP patients carried a transcriptional program enriched for immune regulation and tissue repair (Figure 6J and Figure S12J). This regulatory, tissue‐supportive program was marked by *IL15*, *SMAD6*, and *VEGFB*, which are linked respectively to cytotoxic‐lymphocyte support, restraint of TGF‐β signaling, and endothelial survival (Figure 6J and Figure S12J) [39, 40, 41]. GO enrichment reinforced the contrast: MKPP monocytes were enriched for reparative and anti‐inflammatory pathways, whereas SKPP monocytes were dominated by proinflammatory and tissue‐injury programs (Figure S12K and Tables S28 and S29). This divergence distinguished the regulatory and tissue‐supportive monocyte program in MKPP from the inflammatory and tissue‐injury‐associated program in SKPP.

## DISCUSSION

*K. pneumoniae* has become a major public‐health threat through its capacity to acquire both antimicrobial resistance and hypervirulence traits [42]. Once host defense falters, colonization can advance to invasive pneumonia and severe systemic illness [43]. Using single‐cell profiling of PBMCs from 100 individuals across the severity spectrum, we show that SKPP is not simply an intensified form of mild infection. Instead, it is a systemic failure of immune coordination, marked by myeloid expansion alongside the contraction and functional disruption of the adaptive lymphocyte compartments.

Together, these findings cast SKPP as a state in which innate inflammation and adaptive immune dysfunction coexist: expanded monocytes supply the cellular basis for inflammatory amplification, while changes in CD4^+^ T cells, CD8^+^ T cells, B cells, and NK cells signal weakened adaptive coordination. Comparable peripheral immune shifts have been described in severe COVID‐19 [44, 45], tuberculosis [46], bacterial pneumonia [15], and scrub typhus [47], raising the possibility that severe infections converge on a shared immune imbalance. Our study extends the previous single‐cell analysis of three patients with CRKP pneumonia, two of whom had underlying malignancies [16], by examining a larger, severity‐stratified cohort designed to minimize confounding from pre‐existing immune dysfunction. This design enabled a more focused assessment of immune programs distinguishing mild from severe KPP.

In SKPP, the hyperinflammatory state was concentrated within expanded classical monocytes rather than spread evenly across immune compartments. This sharpens the picture of SKPP, moving it from a nonspecific cytokine elevation to a monocyte‐centered inflammatory program. The coordinated increase in *S100A8/A9/A12*, together with higher *TLR4* and *MYD88* expression and NF‐κB‐related activity, fits a model in which monocyte‐derived *S100* alarmins reinforce innate inflammatory signaling through the *TLR4–MYD88* axis [48, 49, 50]. The present data do not establish causality, yet the convergence of single‐cell transcriptional profiles, plasma protein measurements, and predicted cell–cell communication places this axis at the center of the inflammatory state seen in severe disease. This *S100A8/A9/A12*‐high monocyte program may also carry clinical relevance. Because it is detectable at both the transcriptional and plasma levels, the *S100* alarmins could help flag patients in a monocyte‐driven hyperinflammatory state. Experimental blockade of *S100‐* or *TLR4‐*dependent signaling has proved protective in models of sepsis and acute lung injury [51, 52], lending support to this pathway as a candidate entry point for host‐directed immunomodulation. Direct functional studies will be needed to establish whether targeting the axis can dampen inflammation without compromising antibacterial defense.

CD4^+^ T cell dysfunction in SKPP did not amount to a simple collapse of adaptive immunity [53, 54]. Severe disease instead redirected CD4^+^ T cells toward a hyperactivated but poorly coordinated state. Th1 cells kept their lineage identity and gained stronger inflammatory, interferon‐response, and cytotoxic programs, yet these gains came with inhibitory‐receptor expression and rising “unhelped CD4 T” features, marking activation with weakened helper function rather than terminal exhaustion. Tregs were likewise expanded and transcriptionally activated, but their regulatory identity was bound to inflammatory and exhaustion‐associated reprogramming, indicating that regulatory engagement is reshaped within the severe inflammatory milieu. The proliferative CD4^+^ T cell subset captured the clearest convergence of these altered programs, blending cell‐cycle activity, TCR signaling, interferon responses, mTOR/MYC/E2F activation, exhaustion‐associated markers, and Treg‐like features. SKPP, then, does not merely curtail CD4^+^ T cell help; it distorts helper immunity into a mixed proliferative, inflammatory, and regulatory state that may erode effective adaptive coordination.

Across the cytotoxic compartment (e.g., conventional CD8^+^ T cells, MAIT cells, γδ T cells, and NK cells), SKPP was defined by a dissociation between cytotoxic activity and durable immune function. These populations were enriched for exhaustion‐associated programs yet held on to high expression of core cytolytic molecules such as *GNLY*, *PRF1*, and *GZMA*. Such a profile does not point to a global functional shutdown. It indicates, rather, a cytotoxic‐exhausted state in which the effector machinery persists but sits within a dysfunctional inflammatory context [55]. Sustained type I interferon signaling may be one source of this pressure, while insufficient CD4^+^ T cell help may further weaken the signals needed to sustain durable cytotoxic responses. Under these conditions, CD8‐lineage and NK cells may be driven toward exhaustion‐associated remodeling while retaining cytotoxic potential. The accompanying apoptotic and inflammatory cell‐death signatures imply that this state is also less durable under severe inflammatory stress. Taken together, this may help explain why SKPP preserves cytotoxic pressure yet fails to establish durable adaptive protection.

Humoral immunity in KPP also showed severity‐associated loss of coordination [56]. In MKPP, preserved transcription of B cell receptor signaling genes (e.g., *CD79A/B* and *BTK*), together with a positive association between circulating Tfh cell and plasma‐cell abundance, was consistent with a coordinated humoral immune state in which antigen recognition, helper input, and plasma cell differentiation stayed broadly coupled. In SKPP, plasma cells and late‐state B cells accumulated more prominently, but this expansion ran alongside weaker Tfh–plasma cell coupling, reduced B cell receptor signaling in naïve and memory B cells, and lower expression of genes for antibody secretion and protein handling. Severe disease was therefore distinguished not by a proportionally stronger plasma cell differentiation program, but by plasma cell accumulation, *IGHA1/A2*‐biased immunoglobulin transcription, and inflammatory remodeling of the non‐plasma B cell compartment. Plasma cell expansion in SKPP may thus fail to translate into a more effective humoral response, reflecting instead a partial uncoupling of terminal B cell differentiation from helper‐coordinated antibody maturation. Direct assessment of antibody specificity, affinity, and protective function will be needed to test this interpretation.

Myeloid remodeling formed the cellular bridge between hyperinflammation and failed immune coordination in SKPP. Severe disease was defined not by stronger innate activation alone, but by a redistribution of myeloid function across distinct cellular compartments. mDCs kept elements of co‐stimulatory contact with naïve CD4^+^ T cells yet showed reduced MHC class II expression, weakened phagocytic and antigen‐presenting programs, and lower *RFXANK/IRF8* activity—signs that antigen capture and processing may be impaired even while T cell contact signals remain detectable [57, 58]. In parallel, mMDSCs expanded and took on immunoregulatory, metabolic, ROS‐related, and TLR‐linked programs, their suppressive signatures tracking multiple features of T cell dysfunction [59, 60]. Similar suppressive myeloid states have been associated with T cell inhibition through arginine depletion [61]. Non‐classical monocytes contributed a complement‐related inflammatory layer through increased *C1Q* expression, whereas classical monocytes showed the sharpest severity‐linked divergence: MKPP monocytes held regulatory and tissue‐supportive programs, while SKPP monocytes turned toward an alarmin‐rich, chemokine‐producing, matrix‐remodeling inflammatory state. This arrangement suggests that SKPP reallocates myeloid function away from antigen presentation and tissue repair and toward inflammatory injury, complement activation, and adaptive immune constraint.

Our study has several limitations. First, the analysis rested on peripheral blood and so could not directly resolve the immune landscape inside the infected lung or alveolar space. Although circulating immune changes offer valuable insight into systemic host responses, paired bronchoalveolar lavage fluid or lung‐tissue profiling will be needed to connect these peripheral programs to local pulmonary immunity. Second, several of the mechanistic links proposed here (e.g., *S100–TLR4* signaling, mMDSC‐associated T cell suppression, and Tfh–B cell uncoupling) were inferred from transcriptomic, plasma protein, and ligand–receptor analyses rather than from direct perturbation experiments. Functional validation in cellular and animal models will therefore be necessary to test these mechanisms. Third, the humoral findings rested on transcriptional programs and did not directly measure antibody specificity, affinity, neutralizing activity, or bacterial clearance capacity. Finally, because part of the cohort was integrated from public datasets, residual cohort heterogeneity cannot be fully excluded despite batch correction and quality control. Future work that integrates paired lung and blood sampling, immune functional assays, and experimental validation will help refine this severity‐associated immune framework for KPP.

## CONCLUSION

This study delineates a distinct peripheral immune state in SKPP rather than simply a more intense form of mild infection. Mild disease held on to the hallmarks of coordinated host defense, whereas severe disease combined monocyte‐centered hyperinflammation, reduced antigen‐presentation programs, immunoregulatory myeloid remodeling, and altered states across CD4^+^ T cells, cytotoxic lymphocytes, and B cells. This integrated immune state brings inflammatory amplification and altered adaptive immune coordination within a single disease framework. The observed *S100A8/A9/A12–TLR4–MYD88* transcriptional pattern, cytotoxic‐lymphocyte dysfunction, altered Tfh–B cell coupling, and severity‐linked myeloid polarization provide testable hypotheses for future mechanistic and translational studies. Together, these findings provide a cellular framework for understanding immune dysregulation in KPP and for guiding future mechanistic studies and evaluations of potential host‐directed interventions.

## METHODS

### Study design and participants

The final analytical cohort included 100 individuals: 54 healthy controls (HCs), 23 patients with mild *K. pneumoniae* pneumonia (MKPP), and 23 patients with severe *K. pneumoniae* pneumonia (SKPP) (Table S1). Sample source, group assignment, and dataset accession information for each individual are listed in Table S1. Among the KPP cases, 31 patients were prospectively recruited from participating clinical centers in Shanxi, China, between May 2025 and September 2025. An additional 15 KPP cases [15, 16], together with 54 HCs [44, 62, 63], were obtained from previously published single‐cell RNA‐seq datasets. All KPP cases were confirmed by polymerase chain reaction (PCR), metagenomic next‐generation sequencing, or microbiological culture, as documented in clinical records or dataset metadata. Pneumonia was diagnosed on the basis of compatible clinical symptoms, including cough, fever, sputum production, or pleuritic chest pain, together with radiographic evidence on chest radiography or computed tomography. To minimize confounding by illness duration when comparing severity groups, we applied a uniform enrollment window: both prospectively recruited and publicly sourced MKPP and SKPP cases were sampled within 24–72 h after hospital admission and after clinical severity had been determined. Mild cases were defined as clinically stable patients who did not require intensive care, whereas severe cases were sampled within the same window, which generally coincided with transfer to the intensive care unit (ICU). This design ensured that both groups were captured during an early, established phase of illness.

Severe pneumonia was defined according to the 2007 Infectious Diseases Society of America/American Thoracic Society (IDSA/ATS) criteria for severe CAP [64]. Patients were classified as having severe pneumonia if they met at least one major criterion or three or more minor criteria. The major criteria were septic shock requiring vasopressor support or respiratory failure requiring mechanical ventilation. The minor criteria were respiratory rate >30 breaths/min, PaO_2_/FiO_2_ ratio <250, multilobar infiltrates, confusion or disorientation, uremia (blood urea nitrogen >20 mg/dL), leukopenia (white blood cell count <4000 cells/µL), thrombocytopenia (platelet count <100,000 cells/µL), hypothermia (core temperature <36.8°C), and hypotension requiring aggressive fluid resuscitation.

Patients were excluded if they had any of the following: (1) age younger than 18 years; (2) viral pneumonia; (3) autoimmune disease; (4) immunosuppression (e.g., corticosteroid or chemotherapy treatment, organ transplantation, hematologic malignancy, or HIV infection with a CD4^+^ T cell count below 200 cells/µL); (5) specified comorbidities (e.g., asthma, chronic obstructive pulmonary disease, cystic fibrosis, or bronchiectasis).

### Single‐cell RNA sequencing and data analysis

For prospectively recruited samples, PBMCs were isolated from fresh peripheral blood, and cell viability was confirmed to be greater than 90% using a Countstar automated cell counter. Single‐cell transcriptomic libraries were prepared using the Chromium Single Cell 3′ Kit v3.1 (10× Genomics; 1000123) and sequenced on the Illumina NovaSeq 6000 platform with 2× 150 bp paired‐end reads. Raw reads were aligned to the GRCh38 human reference genome and quantified via kallisto/bustools (kb v0.24.4) [65].

Quality control, normalization, dimensionality reduction, and clustering were performed in Scanpy (v1.11.4). Putative doublets were identified separately for each sample using Scrublet (v0.2.3) [66], with an expected doublet rate of 5% and a score threshold of 0.20. Cells with 5,000 or more detected genes or a mitochondrial gene proportion of 10% or greater were excluded. Gene‐expression counts were library‐size normalized to 10,000 counts per cell and log1p‐transformed. To support robust integration across datasets, a consensus set of 1500 highly variable genes (HVGs) was selected based on their recurrence across samples. Ribosomal, mitochondrial, and immunoglobulin genes were excluded from the HVG set to prevent highly abundant gene families from dominating dimensionality reduction. Principal component analysis (PCA) was performed using the resulting HVG set. The resulting embeddings were corrected for batch effects with Harmony, using sample and dataset as covariates (theta = 2.5 and 1.5, respectively). A k‐nearest‐neighbour graph was constructed from the first 20 Harmony‐corrected principal components using 10 nearest neighbours. Cells were clustered on the batch‐corrected manifold using the Louvain algorithm, and cluster‐specific marker genes were identified with sc.tl.rank_genes_groups.

### Cell clustering and annotations

A shared nearest‐neighbor graph was constructed from the Harmony‐corrected principal components, and cells were clustered using the Louvain algorithm implemented in Scanpy (sc.tl.louvain) at a resolution of 2.0. Major cell identities were assigned by integrating canonical marker‐gene expression with established annotation frameworks from previous single‐cell studies [67, 68]. Eleven major cell populations were identified: plasma cells, B cells, CD4^+^ T cells, CD8^+^ T cells, mucosal‐associated invariant T (MAIT) cells, γδ T cells, natural killer (NK) cells, dendritic cells (DCs), double‐negative T cells (DNTs), monocytes, and megakaryocytes (Figure S3). Cell subtypes were then manually annotated using canonical marker genes and supported by differentially expressed genes identified with sc.tl.rank_genes_groups.

Hierarchical clustering of the 37 immune cell subtypes was performed using subtype‐averaged expression profiles of HVGs. Pairwise Pearson correlation coefficients (*r*) were converted to distances (*d*) using d=(1−r)/2, followed by hierarchical clustering using the ward.D method. Cell‐state connectivity was inferred using PAGA in Scanpy. Differentiation trajectories and pseudotime relationships were reconstructed using Palantir (v1.4.4), which models cell‐state transitions based on diffusion maps. Differentially expressed genes (DEGs) were identified with sc.tl.rank_genes_groups by comparing clusters or disease conditions using the Wilcoxon rank‐sum test with Benjamini‐Hochberg correction. Significantly upregulated genes were defined as those with an adjusted *p* < 0.01 and a log_2_FC > 1.5. Ligand‐receptor interactions were inferred using CellPhoneDB (v5.0.1), with significant interactions defined by permutation testing at *p* < 0.05. Gene regulatory networks were reconstructed with pySCENIC (v0.12.1) to estimate transcription factor regulon activity across cell populations, and regulon activity scores were compared across disease conditions.

### Analysis of immune cell proportions

Differences in the relative abundance of immune cell populations and subtypes across disease conditions were assessed using the Kruskal–Wallis test. For each population, post‐hoc pairwise comparisons were performed for SKPP versus HC, MKPP versus HC, and SKPP versus MKPP, with Bonferroni correction to control the family‐wise error rate. The ratio of observed to expected cell numbers (*R*_*O/E*_) was calculated to quantify the enrichment or depletion of each cell population under each disease condition [69].

### Assessment of cell state scores

Gene set scoring was used to compare cell‐type‐specific activation states across disease conditions. Gene sets were curated from published studies (Tables S3 and S4). For each gene set, scores were calculated at the single‐cell level using sc.tl.score_genes in Scanpy. To account for inter‐sample variation and avoid pseudoreplication, single‐cell scores were aggregated at the sample level by averaging scores across cells of the same cell population within each sample. Statistical comparisons among groups were performed using the Kruskal–Wallis test, followed by Bonferroni‐corrected pairwise post‐hoc comparisons.

### Plasma inflammatory mediator quantification

Plasma samples were obtained from five additional healthy volunteers and from 13 MKPP and 14 SKPP participants who were also included in the single‐cell RNA‐seq cohort. The healthy volunteers were included only in the enzyme‐linked immunosorbent assay (ELISA) analysis. All samples were collected under the approved study protocol with written informed consent, and plasma was isolated from peripheral blood by centrifugation. The S100A8/A9 heterodimer was measured using the Human S100‐A8/A9 Complex ELISA Kit (Catalog No. EH62RB; Thermo, Shanghai, China), and S100A12 was measured using the Human EN‑RAGE/S100A12 ELISA Kit (Catalog No. EH170RB; Thermo, Shanghai, China). All procedures were performed in strict accordance with the manufacturer's instructions. For each analyte, all HC, MKPP, and SKPP samples were assayed on the same 96‐well plate. Each sample and standard was assayed in duplicate, and the entire experiment was independently repeated three times to ensure reproducibility and reliability of the results.

### Flow cytometry

Flow cytometry was performed on PBMCs from eight SKPP participants (SKPP05–SKPP06, SKPP08, and SKPP10–SKPP14) who were also included in the single‐cell sequencing cohort. PBMCs were isolated from participant blood samples and resuspended in RPMI‐1640 medium (Gibco, Invitrogen) supplemented with 10% human serum, 2 mM l‐glutamine, and 10 μg/mL penicillin‐streptomycin. The cells were incubated on ice for 30 min and subsequently stained with fluorescently labeled antibodies for surface or intracellular markers. The staining procedure was also performed on ice in the dark for 30 min. After staining, cells were washed twice with PBS and resuspended in an appropriate volume of PBS for analysis. Flow cytometric data were acquired on a BD LSR‐II flow cytometer using FACSDiva software and analyzed with FlowJo software. The following human monoclonal antibodies were purchased from BD Biosciences (USA): APC‐Cy7‐conjugated anti‐human CD45 (clone: 2D1), BV480‐conjugated anti‐human CD4 (clone: L120), FITC‐conjugated anti‐human CD8 (clone: RPA‐T8), BV605‐conjugated anti‐human CD25 (clone: 2A3), PE‐conjugated anti‐human FoxP3 (clone: 259D/C7), BV421‐conjugated anti‐human CD279 (PD‐1) (clone: EH12.1), and APC‐conjugated anti‐human CD152 (CTLA‐4) (clone: BNI3). All antibodies and dyes were used according to the manufacturer's recommended protocols.

### Statistical analysis

Statistical analyses and data visualization were performed using Python and R. Statistical significance is indicated as follows: ns, not significant, *p* > 0.05; *p < 0.05; **p < 0.01; ***p < 0.001.

## AUTHOR CONTRIBUTIONS

**Hongquan Chen**: Conceptualization; methodology; software; data curation; investigation; validation; formal analysis; visualization; project administration; writing—original draft; writing—review and editing. **Xiaoling Wang**: Methodology; data curation; investigation; supervision; project administration; resources. **Xiang Liu**: Methodology; data curation; investigation; supervision; project administration; resources. **Kui Dong**: Methodology; data curation; supervision; project administration; resources. **Yanying Feng**: Investigation; validation. **Min Yuan**: Validation. **Shiyi Wang**: Validation. **Fangfang Fan**: Investigation. **Yong Tian**: Investigation. **Zhiming Kang**: Investigation. **Xiushuai Du**: Software. **Chaolu Hasi**: Resources. **Hong Lu**: Investigation. **Wei Guan**: Resources. **Xu Chen**: Investigation. **Mengyu Cheng**: Resources. **Wei Wei**: Investigation. **Nan Guo**: Investigation. **Zhiguo Liu**: Investigation. **Xiaotong Qiu**: Investigation. **Yiwei Shi**: Supervision; project administration; resources; writing—review and editing. **Kun Xiao**: Supervision; funding acquisition; project administration; resources; writing—review and editing. **Yi Wang**: Conceptualization; methodology; software; data curation; supervision; funding acquisition; project administration; resources; writing—original draft; writing—review and editing. **Zhenjun Li**: Conceptualization; methodology; software; data curation; supervision; funding acquisition; project administration; resources; writing—original draft; writing—review and editing.

## CONFLICT OF INTEREST STATEMENT

The authors declare no conflicts of interest.

## ETHICS STATEMENT

The ethical approval (No. ICDC‐LPJ‐2024006) for this study was obtained from the Ethics Committee of National Institute for Communicable Disease Control and Prevention, Chinese Center for Disease Control and Prevention.

## Supporting information


**Figure S1:** Distribution of clinical characteristics in the integrated dataset.
**Figure S2:** Single‐cell transcriptional profiling of peripheral blood mononuclear cells from 100 subjects, related to Figure 1.
**Figure S3:** Clustering quality and immune cell alterations in peripheral blood mononuclear cells from healthy donors and *Klebsiella pneumoniae* pneumonia patients, related to Figure 1.
**Figure S4:** Characterization of hyperinflammatory cell subtypes across disease severity groups, related to Figure 2.
**Figure S5:** Identification of hyperinflammatory subtypes associated with potential inflammatory responses in peripheral blood mononuclear cells, related to Figure 2.
**Figure S6:** Characterization of hyperinflammatory subtypes associated with potential inflammatory responses in peripheral blood mononuclear cells, related to Figure 2.
**Figure S7:** Extended characterization of CD4^+^ T cell states across disease conditions, related to Figure 3.
**Figure S8:** Flow cytometry plots showing gating strategy in CD4^+^ T cells from patients with severe *Klebsiella pneumoniae* pneumonia, related to Figure 3.
**Figure S9:** Extended characterization of CD8^+^ T cell states across disease conditions, related to Figure 4.
**Figure S10:** Flow cytometry plots showing gating strategy in CD8^+^ T cells from patients with severe *Klebsiella pneumoniae* pneumonia, related to Figure 4.
**Figure S11:** Extended characterization of B cell states across disease conditions, related to Figure 5.
**Figure S12:** Extended characterization of myeloid cell states across disease conditions, related to Figure 6.
**Figure S13:** Bar chart showing the composition of *C1QA/B/C* scores, broken down by relative contributions from each cell subtype.

**Table S1:** The laboratory findings and clinical features of enrolled *Klebsiella pneumoniae* pneumonia patients.
**Table S2:** Marker genes and signature genes for cell subtypes, related to Figure 1 and Figure S3.
T**able S3:** Inflammatory genes and cytokine score, related to Figure 2 and Figures S4–S6.
**Table S4:** Signature genes used to define functional status in immune cells.
**Table S5:** Differentially expressed genes in proliferative CD4^+^ T cells between patients with mild disease and healthy controls.
**Table S6:** Differentially expressed genes in proliferative CD4^+^ T cells between patients with severe disease and healthy controls.
**Table S7:** Differentially expressed genes in proliferative CD4^+^ T cells between patients with severe and mild disease.
**Table S8:** Gene set enrichment analysis of proliferative CD4^+^ T cells between patients with mild disease and healthy controls.
**Table S9:** Gene set enrichment analysis of proliferative CD4^+^ T cells between patients with severe disease and healthy controls.
**Table S10:** Gene set enrichment analysis of proliferative CD4^+^ T cells between patients with severe and mild disease.
**Table S11:** Gene Ontology enrichment analysis of significantly upregulated genes in Cluster 4.
**Table S12:** Significantly upregulated genes in CD8^+^ T cells from patients with mild disease compared with healthy controls.
**Table S13:** Significantly upregulated genes in CD8^+^ T cells from patients with severe disease compared with healthy controls.
**Table S14:** Gene set enrichment analysis of CD8^+^ T cells between patients with mild disease and healthy controls.
**Table S15:** Gene set enrichment analysis of CD8^+^ T cells between patients with severe disease and healthy controls.
**Table S16:** Gene set enrichment analysis of CD8^+^ T cells between patients with severe and mild disease.
**Table S17:** Significantly upregulated genes in naïve and memory B cells from patients with mild disease compared with healthy controls.
**Table S18:** Significantly upregulated genes in naïve and memory B cells from patients with severe disease compared with healthy controls.
**Table S19:** Gene Ontology enrichment analysis of genes significantly upregulated in naïve and memory B cells from patients with mild disease compared with healthy controls.
**Table S20:** Gene Ontology enrichment analysis of genes significantly upregulated in naïve and memory B cells from patients with severe disease compared with healthy controls.
**Table S21:** Sample‐level correlations between mMDSC suppressive scores and T‐cell functional scores.
**Table S22:** Significantly upregulated genes in monocytic myeloid‐derived suppressor cells from patients with mild disease compared with healthy controls.
**Table S23:** Significantly upregulated genes in monocytic myeloid‐derived suppressor cells from patients with severe disease compared with healthy controls.
**Table S24:** Gene Ontology enrichment analysis of genes significantly upregulated in monocytic myeloid‐derived suppressor cells from patients with mild disease compared with healthy controls.
**Table S25:** Gene Ontology enrichment analysis of genes significantly upregulated in monocytic myeloid‐derived suppressor cells from patients with severe disease compared with healthy controls.
**Table S26:** Significantly upregulated genes in classical monocytes from patients with mild disease compared with healthy controls.
**Table S27:** Significantly upregulated genes in classical monocytes from patients with severe disease compared with healthy controls.
**Table S28:** Gene Ontology enrichment analysis of genes significantly upregulated in classical monocytes from patients with mild disease compared with healthy controls.
**Table S29:** Gene Ontology enrichment analysis of genes significantly upregulated in classical monocytes from patients with severe disease compared with healthy controls.

## Data Availability

The data that support the findings of this study are openly available in OMIX, National Genomics Data Center (NGDC), China National Center for Bioinformation at https://ngdc.cncb.ac.cn/omix/release/OMIX014353, reference number OMIX014353. The sequencing data from the 31 newly included samples reported in this paper have been deposited in the OMIX, China National Center for Bioinformation/Beijing Institute of Genomics, Chinese Academy of Sciences (https://ngdc.cncb.ac.cn/omix/release/OMIX014353). A total of 15 KPP cases were included in this study, with single‐cell datasets from 14 cases obtained from the OMIX database (accession no. OMIX010124, available at https://ngdc.cncb.ac.cn/omix/release/OMIX010124) and the remaining one from the GEO database (accession no. GSE263380, available at https://www.ncbi.nlm.nih.gov/geo/query/acc.cgi?acc=GSE263380). Additionally, 54 healthy control samples were sourced from the following three publicly available resources: the Zenodo repository (10.5281/zenodo.17114800), the Peking University COVID‐19 Single Cell Database (http://covid19.cancer-pku.cn), and the GEO database (accession no. GSE165080, available at https://www.ncbi.nlm.nih.gov/geo/query/acc.cgi?acc=GSE165080). Custom scripts used for data analysis and figure generation are publicly available at https://github.com/bio806/KPP-single-cell-analysis. Supplementary materials (figures, tables, graphical abstract, slides, videos, Chinese translated version, and update materials) may be found in the online DOI or iMeta Science http://www.imeta.science/.

## References

[1] Ma, Zheng , Li Mo , Chunhong Li , Jihua Hu , Wenyu Zheng , Shaohui Zeng , Bohai Yu , and Shuo Jiang . 2025. “Epidemiology and Genomic Characteristics of Carbapenem‐Resistant *Klebsiella pneumoniae* in Intensive Care Unit From 2021 to 2024.” BMC Microbiology 25: 774. 10.1186/s12866-025-04497-0 PMC12642057 41287044

[2] Liu, Yan‐Ning , Yun‐Fa Zhang , Qiang Xu , Yan Qiu , Qing‐Bin Lu , Tao Wang , Xiao‐Ai Zhang , et al. 2023. “Infection and Co‐Infection Patterns of Community‐Acquired Pneumonia in Patients of Different Ages in China From 2009 to 2020: a National Surveillance Study.” The Lancet Microbe 4: e330–e339. 10.1016/s2666-5247(23)00031-9 PMC12514336 37001538

[3] Juan, Chih‐Han , Shih‐Yu Fang , Chia‐Hsin Chou , Tsung‐Ying Tsai , and Yi‐Tsung Lin . 2020. “Clinical Characteristics of Patients With Pneumonia Caused by *Klebsiella pneumoniae* in Taiwan and Prevalence of Antimicrobial‐Resistant and Hypervirulent Strains: A Retrospective Study.” Antimicrobial Resistance & Infection Control 9: 4. 10.1186/s13756-019-0660-x PMC6942382 31911832

[4] Song, Jae‐Hoon , Won Sup Oh , Cheol‐In Kang , Doo Ryeon Chung , Kyong Ran Peck , Kwan Soo Ko , Joon Sup Yeom , et al. 2008. “Epidemiology and Clinical Outcomes of Community‐Acquired Pneumonia in Adult Patients in Asian Countries: A Prospective Study by the Asian Network for Surveillance of Resistant Pathogens.” International Journal of Antimicrobial Agents 31: 107–114. 10.1016/j.ijantimicag.2007.09.014 PMC7134693 18162378

[5] Gu, Danxia , Ning Dong , Zhiwei Zheng , Di Lin , Man Huang , Lihua Wang , Edward Wai‐Chi Chan , et al. 2018. “A Fatal Outbreak of ST11 Carbapenem‐Resistant Hypervirulent *Klebsiella pneumoniae* in a Chinese Hospital: A Molecular Epidemiological Study.” The Lancet Infectious Diseases 18: 37–46. 10.1016/s1473-3099(17)30489-9 28864030

[6] Wu, Yuchen , Fan Pu , Zelin Yan , Yanyan Zhang , Kaichao Chen , Shengkai Li , Yuezhuo Wang , et al. 2025. “Geographic Containment and Virulence‐Resistance Trade‐Offs Drive the Evolution of Hypervirulent *Klebsiella pneumoniae* .” iMeta 4: e70077. 10.1002/imt2.70077 PMC12528002 41112046

[7] Luo, Hao , Yao Wang , Huiyu Hou , Junbo Yang , and Yong‐Xin Liu . 2026. “Advances and Applications in Sequencing‐Based Pathogen Surveillance.” aBIOTECH 7: 100004. 10.1016/j.abiote.2025.100004 PMC12973409 41940150

[8] Chang, De , Lokesh Sharma , Charles S. Dela Cruz , and Dong Zhang . 2021. “Clinical Epidemiology, Risk Factors, and Control Strategies of *Klebsiella pneumoniae* Infection.” Frontiers in Microbiology 12: 750662. 10.3389/fmicb.2021.750662 PMC8724557 34992583

[9] Opoku‐Temeng, Clement , Natalia Malachowa , Scott D. Kobayashi , and Frank R. DeLeo . 2022. “Innate Host Defense Against *Klebsiella pneumoniae* and the Outlook for Development of Immunotherapies.” Journal of Innate Immunity 14: 167–181. 10.1159/000518679 PMC9149408 34628410

[10] Tang, Bingxin , Wenwen Cui , Xiao Li , and Huan Yang . 2024. “ Salmonella–Host–Microbiota Interactions: Immunity and Metabolism .” iMetaOmics 1: e16. 10.1002/imo2.16 PMC12806413 41675554

[11] Jiang, Fei , Jiebang Jiang , Wenping He , Guokai Dong , Nana Xu , Li Meng , Yunyun Zhao , et al. 2023. “Chrysophanol Alleviates Acute Lung Injury Caused by *Klebsiella pneumoniae* Infection by Inhibiting Pro‐Inflammatory Cytokine Production.” Phytotherapy Research 37: 2965–2978. 10.1002/ptr.7792 36879546

[12] Yu, Wei , Linyan Zeng , Xiang Lian , Lushun Jiang , Hao Xu , Wenhui Guo , Beiwen Zheng , and Yonghong Xiao . 2024. “Dynamic Cytokine Profiles of Bloodstream Infection Caused by *Klebsiella pneumoniae* in China.” Annals of Clinical Microbiology and Antimicrobials 23: 79. 10.1186/s12941-024-00739-7 PMC11344948 39182143

[13] Hotchkiss, Richard S. , Guillaume Monneret , and Didier Payen . 2013. “Sepsis‐Induced Immunosuppression: From Cellular Dysfunctions to Immunotherapy.” Nature Reviews Immunology 13: 862–874. 10.1038/nri3552 PMC4077177 24232462

[14] Bengoechea, José A , and Joana Sa Pessoa . 2018. “ *Klebsiella pneumoniae* Infection Biology: Living to Counteract Host Defences.” FEMS Microbiology Reviews 43: 123–144. 10.1093/femsre/fuy043 PMC6435446 30452654

[15] Xiao, Kun , Yan Cao , Peng Yan , Ye Hu , Laurence Don Wai Luu , Pan Pan , Hongjun Gu , et al. 2025. “A Large‐Scale Single‐Cell Atlas Reveals the Peripheral Immune Panorama of Bacterial Pneumonia.” American Journal of Respiratory and Critical Care Medicine 211: 2363–2381. 10.1164/rccm.202501-0217OC PMC12700268 40758632

[16] Sun, Yajiao , Fuhui Chen , Hui Ma , Dongjie Wang , Dong Wang , Jingwen Zhang , Zhe, Jiang , et al. 2024. “Exploring the Immune Characteristions of CRKP Pneumonia at Single‐Cell Level.” Computers in Biology and Medicine 177: 108574. 10.1016/j.compbiomed.2024.108574 38772102

[17] Zhang, Shengwei , Nan Zhang , Jing Han , Zeyu Sun , Hua Jiang , Wenhua Huang , Decong Kong , et al. 2024. “Dynamic Immune Status Analysis of Peripheral Blood Mononuclear Cells in Patients With *Klebsiella pneumoniae* Bloodstream Infection Sepsis Using Single‐Cell RNA Sequencing.” Frontiers in Immunology 15: 1380211. 10.3389/fimmu.2024.1380211 PMC11185935 38898888

[18] Liu, Wendao , Johnathan Jia , Yulin Dai , Wenhao Chen , Guangsheng Pei , Qiheng Yan , and Zhongming Zhao . 2022. “Delineating COVID‐19 Immunological Features Using Single‐Cell RNA Sequencing.” The Innovation 3: 100289. 10.1016/j.xinn.2022.100289 PMC9299978 35879967

[19] Xiao, Kun , Yan Cao , Zhihai Han , Yuxiang Zhang , Laurence Don Wai Luu , Liang Chen , Peng Yan , et al. 2025. “A Pan‐Immune Panorama of Bacterial Pneumonia Revealed by a Large‐Scale Single‐Cell Transcriptome Atlas.” Signal Transduction and Targeted Therapy 10: 5. 10.1038/s41392-024-02093-8 PMC11701081 39757231

[20] Wang, Xiaoqing , Ying Luo , Qiang Zhou , and Jie Ma . 2025. “The Roles of S100A8/A9 and S100A12 in Autoimmune Diseases: Mechanisms, Biomarkers, and Therapeutic Potential.” Autoimmunity Reviews 24: 103920. 10.1016/j.autrev.2025.103920 40876560

[21] Zhou, Liang , Mark M. W. Chong , and Dan R. Littman . 2009. “Plasticity of CD4+ T Cell Lineage Differentiation.” Immunity 30: 646–655. 10.1016/j.immuni.2009.05.001 19464987

[22] Mucida, Daniel , Mohammad Mushtaq Husain , Sawako Muroi , Femke van Wijk , Ryo Shinnakasu , Yoshinori Naoe , Bernardo Sgarbi Reis , et al. 2013. “Transcriptional Reprogramming of Mature CD4+ Helper T Cells Generates Distinct MHC Class II–Restricted Cytotoxic T Lymphocytes.” Nature Immunology 14: 281–289. 10.1038/ni.2523 PMC3581083 23334788

[23] Dong, Chen . 2021. “Cytokine Regulation and Function in T Cells.” Annual Review of Immunology 39: 51–76. 10.1146/annurev-immunol-061020-053702 33428453

[24] Kaech, Susan M. , and Weiguo Cui . 2012. “Transcriptional Control of Effector and Memory CD8+ T Cell Differentiation.” Nature Reviews Immunology 12: 749–761. 10.1038/nri3307 PMC4137483 23080391

[25] Wang, Yi , Laurence Don Wai Luu , Shuang Liu , Xiong Zhu , Siyuan Huang , Fang Li , Xiaolan Huang , et al. 2023. “Single‐Cell Transcriptomic Analysis Reveals a Systemic Immune Dysregulation in COVID‐19‐Associated Pediatric Encephalopathy.” Signal Transduction and Targeted Therapy 8: 398. 10.1038/s41392-023-01641-y PMC10582072 37848421

[26] Cillo, Anthony R. , Carly Cardello , Feng Shan , Lilit Karapetyan , Sheryl Kunning , Cindy Sander , Elizabeth Rush , et al. 2024. “Blockade of LAG‐3 and PD‐1 Leads to Co‐Expression of Cytotoxic and Exhaustion Gene Modules in CD8+ T Cells to Promote Antitumor Immunity.” Cell 187: 4373–4388.e15. 10.1016/j.cell.2024.06.036 PMC11346583 39121849

[27] Fernandes, Ricardo A. , Leon Su , Yoko Nishiga , Junming Ren , Aladdin M. Bhuiyan , Ning Cheng , Calvin J. Kuo , et al. 2020. “Immune Receptor Inhibition Through Enforced Phosphatase Recruitment.” Nature 586: 779–784. 10.1038/s41586-020-2851-2 PMC7875542 33087934

[28] Wherry, E. John , and Makoto Kurachi . 2015. “Molecular and Cellular Insights Into T Cell Exhaustion.” Nature Reviews Immunology 15: 486–499. 10.1038/nri3862 PMC4889009 26205583

[29] Oh, Ji Eun , Eric Song , Miyu Moriyama , Patrick Wong , Sophia Zhang , Ruoyi Jiang , Shirin Strohmeier , et al. 2021. “Intranasal Priming Induces Local Lung‐Resident B Cell Populations That Secrete Protective Mucosal Antiviral IgA.” Science Immunology 6: eabj5129. 10.1126/sciimmunol.abj5129 PMC8762609 34890255

[30] Shulman, Ziv , Alexander D. Gitlin , Jason S. Weinstein , Begoña Lainez , Enric Esplugues , Richard A. Flavell , Joseph E. Craft , and Michel C. Nussenzweig . 2014. “Dynamic Signaling by T Follicular Helper Cells during Germinal Center B Cell Selection.” Science 345: 1058–1062. 10.1126/science.1257861 PMC4519234 25170154

[31] Qin, Guohui , Shasha Liu , Li Yang , Weina Yu , and Yi Zhang . 2021. “Myeloid Cells in COVID‐19 Microenvironment.” Signal Transduction and Targeted Therapy 6: 372. 10.1038/s41392-021-00792-0 PMC8549428 34707085

[32] Jin, Fangfang , Lingxiang Xie , Hongqi Zhang , Xiang Fan , Jiaxing Tian , Wei Liu , Yang Xiao , and Xinrong Fan . 2025. “Dendritic Cells: Origin, Classification, Development, Biological Functions, and Therapeutic Potential.” MedComm 6: e70455. 10.1002/mco2.70455 PMC12587171 41200280

[33] Zhao, Fei , Bastian Hoechst , Austin Duffy , Jaba Gamrekelashvili , Suzanne Fioravanti , Michael P. Manns , Tim F. Greten , and Firouzeh Korangy . 2012. “S100A9 a New Marker for Monocytic Human Myeloid‐Derived Suppressor Cells.” Immunology 136: 176–183. 10.1111/j.1365-2567.2012.03566.x PMC3403264 22304731

[34] Li, Jieyao , Liping Wang , Xinfeng Chen , Lifeng Li , Yu Li , Yu Ping , Lan Huang , et al. 2017. “CD39/CD73 Upregulation on Myeloid‐Derived Suppressor Cells via TGF‐β‐mTOR‐HIF‐1 Signaling in Patients With Non‐Small Cell Lung Cancer.” OncoImmunology 6: e1320011. 10.1080/2162402x.2017.1320011 PMC5486179 28680754

[35] Azoulay, Elie , Julien Zuber , Ahmed Aziz Bousfiha , Yun Long , Ying Tan , Sushan Luo , Meriem Essafti , and Djillali Annane . 2024. “Complement System Activation: Bridging Physiology, Pathophysiology, and Therapy.” Intensive Care Medicine 50: 1791–1803. 10.1007/s00134-024-07611-4 39254734

[36] Liang, Yafeng , Nengli Yang , Guoquan Pan , Bingxin Jin , Shufen Wang , and Wei Ji . 2018. “Elevated IL‐33 Promotes Expression of MMP2 and MMP9 via Activating STAT3 in Alveolar Macrophages during LPS‐Induced Acute Lung Injury.” Cellular & Molecular Biology Letters 23: 52. 10.1186/s11658-018-0117-x PMC6208075 30410547

[37] Nakata, Kentaro , Mikio Okazaki , Tomohisa Sakaue , Rie Kinoshita , Yuhei Komoda , Dai Shimizu , Haruchika Yamamoto , et al. 2022. “Functional Blockage of S100A8/A9 Ameliorates Ischemia–Reperfusion Injury in the Lung.” Bioengineering 9: 673. 10.3390/bioengineering9110673 PMC9687586 36354584

[38] Chakraborty, Deblina , Stefanie Zenker , Jan Rossaint , Anna Hölscher , Michele Pohlen , Alexander Zarbock , Johannes Roth , and Thomas Vogl . 2017. “Alarmin S100A8 Activates Alveolar Epithelial Cells in the Context of Acute Lung Injury in a TLR4‐Dependent Manner.” Frontiers in Immunology 8: 1493. 10.3389/fimmu.2017.01493 PMC5693860 29180999

[39] Mura, Marco , Bing Han , Cristiano F. Andrade , Rashmi Seth , David Hwang , Thomas K. Waddell , Shaf Keshavjee , and Mingyao Liu . 2006. “The Early Responses of VEGF and Its Receptors during Acute Lung Injury: Implication of VEGF in Alveolar Epithelial Cell Survival.” Critical Care 10: R130. 10.1186/cc5042 PMC1751039 16968555

[40] Kandikattu, Hemanth Kumar , Sathisha Upparahalli Venkateshaiah , Sandeep, Kumar , and Anil Mishra . 2020. “IL‐15 Immunotherapy Is a Viable Strategy for COVID‐19.” Cytokine & Growth Factor Reviews 54: 24–31. 10.1016/j.cytogfr.2020.06.008 PMC7537239 32536564

[41] Zhang, T. , J. Wu , N. Ungvijanpunya , O. Jackson‐Weaver , Y. Gou , J. Feng , T. V. Ho , et al. 2018. “Smad6 Methylation Represses NFκb Activation and Periodontal Inflammation.” Journal of Dental Research 97: 810–819. 10.1177/0022034518755688 PMC6728583 29420098

[42] Yang, Xuemei , Ning Dong , Edward Wai‐Chi Chan , Rong Zhang , and Sheng Chen . 2021. “Carbapenem Resistance‐Encoding and Virulence‐Encoding Conjugative Plasmids in *Klebsiella pneumoniae* .” Trends in Microbiology 29: 65–83. 10.1016/j.tim.2020.04.012 32448764

[43] Paczosa, Michelle K. , and Joan Mecsas . 2016. “ *Klebsiella pneumoniae*: Going on the Offense With a Strong Defense.” Microbiology and Molecular Biology Reviews 80: 629–661. 10.1128/MMBR.00078-15 PMC4981674 27307579

[44] Ren, Xianwen , Wen Wen , Xiaoying Fan , Wenhong Hou , Bin Su , Pengfei Cai , Jiesheng Li , et al. 2021. “COVID‐19 Immune Features Revealed by a Large‐Scale Single‐Cell Transcriptome Atlas.” Cell 184: 1895–1913.e19. 10.1016/j.cell.2021.01.053 PMC7857060 33657410

[45] Ren, Lili , Yiwei Liu , Yeming Wang , Chao Wu , Li Guo , Lan Chen , Xinming Wang , et al. 2025. “Longitudinal Landscape of Immune Reconstitution After Acute SARS‐CoV‐2 Infection at Single‐Cell Resolution.” Science Bulletin 70: 323–327. 10.1016/j.scib.2024.07.011 39107149

[46] Wang, Yi , Maike Zheng , Yun Zhang , Yu Xue , Sibo Long , Chaohong Wang , Qing Sun , et al. 2025. “Single‐Cell Transcriptomic Analysis of the Immune Response to COVID‐19 and Tuberculosis Coinfection.” Exploration 5: 20240022. 10.1002/exp.20240022 PMC12561472 41163794

[47] Guo, Yijia , Yi Niu , Nihui Zhang , Xiao Han , Liyuan Zhang , Yongguo Du , Lifen Han , et al. 2026. “Single‐Cell Profiling of Peripheral Blood Reveals Acute‐Phase Immune Dysregulation in Human Scrub Typhus.” iMeta 5: e70167. 10.1002/imt2.70167 PMC13442654 42564233

[48] Zhou, Lvzhou , Yao Xiao , Jinlian He , Hong Ren , Caiping Zhao , Chuanhai Zhang , Xiao Shu , et al. 2025. “Euchrenone A10 Attenuates Septic Lung Injury Though S100A8/A9‐Dependent TLR4/MyD88/NF‐κB Signaling.” Phytomedicine 148: 157336. 10.1016/j.phymed.2025.157336 41038141

[49] Vogl, Thomas , Klaus Tenbrock , Stephan Ludwig , Nadja Leukert , Christina Ehrhardt , Marieke A. D. van Zoelen , Wolfgang Nacken , et al. 2007. “Mrp8 and Mrp14 Are Endogenous Activators of Toll‐Like Receptor 4, Promoting Lethal, Endotoxin‐Induced Shock.” Nature Medicine 13: 1042–1049. 10.1038/nm1638 17767165

[50] Wang, Qian , Gangyu Long , Hong Luo , Xiqun Zhu , Yang Han , You Shang , Dingyu Zhang , and Rui Gong . 2023. “S100A8/A9: an Emerging Player in Sepsis and Sepsis‐Induced Organ Injury.” Biomedicine & Pharmacotherapy 168: 115674. 10.1016/j.biopha.2023.115674 37812889

[51] Zhao, Boying , Renfu Lu , Jianjun Chen , Ming Xie , Xingji Zhao , and Lingwen Kong . 2021. “S100A9 Blockade Prevents Lipopolysaccharide‐Induced Lung Injury via Suppressing the NLRP3 Pathway.” Respiratory Research 22: 45. 10.1186/s12931-021-01641-y PMC7866705 33549095

[52] Chen, Sini , Jingying Lin , Jiahao Huang , Liehua Deng , and Yuanli Zhang . 2026. “The S100A8/A9–NETosis Feedback Loop in Sepsis: Potential Mechanisms, Immune Crosstalk, and Therapeutic Targeting.” Frontiers in Immunology 17: 1790788. 10.3389/fimmu.2026.1790788 PMC13132756 42079585

[53] Martin, Matthew D. , Vladimir P. Badovinac , and Thomas S. Griffith . 2020. “CD4 T Cell Responses and the Sepsis‐Induced Immunoparalysis State.” Frontiers in Immunology 11: 1364. 10.3389/fimmu.2020.01364 PMC7358556 32733454

[54] Jensen, Isaac J. , Frances V. Sjaastad , Thomas S. Griffith , and Vladimir P. Badovinac . 2018. “Sepsis‐Induced T Cell Immunoparalysis: The Ins and Outs of Impaired T Cell Immunity.” The Journal of Immunology 200: 1543–1553. 10.4049/jimmunol.1701618 PMC5826615 29463691

[55] Chang, Katherine , Catherine Svabek , Cristina Vazquez‐Guillamet , Bryan Sato , David Rasche , Strother Wilson , Paul Robbins , et al. 2014. “Targeting the Programmed Cell Death 1: Programmed Cell Death Ligand 1 Pathway Reverses T Cell Exhaustion in Patients With Sepsis.” Critical Care 18: R3. 10.1186/cc13176 PMC4056005 24387680

[56] Davies, Kate , and James E. McLaren . 2024. “Destabilisation of T Cell‐Dependent Humoral Immunity in Sepsis.” Clinical Science 138: 65–85. 10.1042/CS20230517 PMC10781648 38197178

[57] Poehlmann, Holger , Joerg C. Schefold , Heidrun Zuckermann‐Becker , Hans‐Dieter Volk , and Christian Meisel . 2009. “Phenotype Changes and Impaired Function of Dendritic Cell Subsets in Patients With Sepsis: A Prospective Observational Analysis.” Critical Care 13: R119. 10.1186/cc7969 PMC2750167 19604380

[58] Grimaldi, D. , S. Louis , F. Pène , G. Sirgo , C. Rousseau , Y. E. Claessens , L. Vimeux , et al. 2011. “Profound and Persistent Decrease of Circulating Dendritic Cells Is Associated With ICU‐Acquired Infection in Patients With Septic Shock.” Intensive Care Medicine 37: 1438–1446. 10.1007/s00134-011-2306-1 21805160

[59] Schrijver, Irene T. , Charlotte Théroude , and Thierry Roger . 2019. “Myeloid‐Derived Suppressor Cells in Sepsis.” Frontiers in Immunology 10: 327. 10.3389/fimmu.2019.00327 PMC6400980 30873175

[60] Dai, Jun , Ajinkya Kumbhare , Dima Youssef , Charles E. McCall , and Mohamed El Gazzar . 2017. “Intracellular S100A9 Promotes Myeloid‐Derived Suppressor Cells during Late Sepsis.” Frontiers in Immunology 8: 1565. 10.3389/fimmu.2017.01565 PMC5698275 29204146

[61] Darcy, Christabelle J. , Gabriela Minigo , Kim A. Piera , Joshua S. Davis , Yvette R. McNeil , Youwei Chen , Alicia D. Volkheimer , et al. 2014. “Neutrophils With Myeloid Derived Suppressor Function Deplete Arginine and Constrain T Cell Function in Septic Shock Patients.” Critical Care 18: R163. 10.1186/cc14003 PMC4261583 25084831

[62] Tian, Huaping , Yuhong Chen , Tujing Zhao , Lin Ye , Hongjing Li , Zheng Li , Wenqiao Qiu , et al. 2025. “Single‐Cell RNA‐seq Reveals Cell Type‐Specific Molecular and Genetic Associations With Primary Open‐Angle Glaucoma.” Signal Transduction and Targeted Therapy 10: 338. 10.1038/s41392-025-02438-x PMC12511369 41068080

[63] Wang, Xiaorui , Han Bai , Junpeng Ma , Hongyu Qin , Qiqi Zeng , Fang Hu , Tingting Jiang , et al. 2022. “Identification of Distinct Immune Cell Subsets Associated With Asymptomatic Infection, Disease Severity, and Viral Persistence in COVID‐19 Patients.” Frontiers in Immunology 13: 812514. 10.3389/fimmu.2022.812514 PMC8905648 35281000

[64] Mandell, Lionel A. , Richard G. Wunderink , Antonio Anzueto , John G. Bartlett , G. Douglas Campbell , Nathan C. Dean , Scott F. Dowell , et al. 2007. “Infectious Diseases Society of America/American Thoracic Society Consensus Guidelines on the Management of Community‐Acquired Pneumonia in Adults.” Clinical Infectious Diseases 44: S27–S72. 10.1086/511159 PMC7107997 17278083

[65] Bray, Nicolas L. , Harold Pimentel , Páll Melsted , and Lior Pachter . 2016. “Near‐Optimal Probabilistic RNA‐seq Quantification.” Nature Biotechnology 34: 525–527. 10.1038/nbt.3519 27043002

[66] Wolock, Samuel L. , Romain Lopez , and Allon M. Klein . 2019. “Scrublet: Computational Identification of Cell Doublets in Single‐Cell Transcriptomic Data.” Cell Systems 8: 281–291.e9. 10.1016/j.cels.2018.11.005 PMC6625319 30954476

[67] Wang, Yi , Siyuan Yang , Bing Han , Xiufang Du , Huali Sun , Yufeng Du , Yinli Liu , et al. 2024. “Single‐Cell Landscape Revealed Immune Characteristics Associated With Disease Phases in Brucellosis Patients.” iMeta 3: e226. 10.1002/imt2.226 PMC11316929 39135683

[68] Wang, Yi , Shuzi Liu , Laurence Don Wai Luu , Yongzhi Zhai , Chenliang Zhu , Zhaomin Feng , Yao Tan , et al. 2026. “A Large‐Scale Single‐Cell Transcriptomic Atlas Indicates the Immune Panorama of Influenza A Infection.” iMeta 5: e70121. 10.1002/imt2.70121 PMC13147933 42099460

[69] Zhang, Lei , Xin Yu , Liangtao Zheng , Yuanyuan Zhang , Yansen Li , Qiao Fang , Ranran Gao , et al. 2018. “Lineage Tracking Reveals Dynamic Relationships of T Cells in Colorectal Cancer.” Nature 564: 268–272. 10.1038/s41586-018-0694-x 30479382

