# Supplementary material for: Monocyte inflammation and adaptive immune dysfunction in severe Klebsiella pneumoniae pneumonia
Source: Imeta. 2026 Sep 21;5(5):e70172. doi: 10.1002/imt2.70172 (PMC13594409; doi:10.1002/imt2.70172)
Supplement: Supplementary file 1 [file IMT2-5-e70172-s002.docx]

**Supporting information to**

**Monocyte inflammation and adaptive immune dysfunction in severe *Klebsiella pneumoniae* pneumonia**

**Running title:** Immune dysregulation in severe *Klebsiella pneumoniae* pneumonia

Hongquan Chen^1#^, Xiaoling Wang^2#^, Xiang Liu^3#^, Kui Dong^4,5#^, Yanying Feng^1,6^, Min Yuan^1^, Shiyi Wang^1^, Fangfang Fan^3^, Yong Tian^2^, Zhiming Kang^4,5^, Xiushuai Du^7^, Chaolu Hasi^8^, Hong Lu^8^, Wei Guan^9^, Xu Chen^9^, Mengyu Cheng^10^, Wei Wei^1^, Nan Guo^1,4^, Zhiguo Liu^1^, Xiaotong Qiu^1^, Yiwei Shi^3*^, Kun Xiao^11*^, Yi Wang^12,13*^, Zhenjun Li^1*^

^1^ National Key Laboratory of Intelligent Tracking and Forecasting for Infectious Diseases, National Institute for Communicable Disease Control and Prevention, Chinese Center for Disease Control and Prevention & Chinese Academy of Preventive Medicine, Beijing, 102200, China.

^2^ Department of Clinical Laboratory, Shanxi Hospital of Traditional Chinese Medicine, Taiyuan, China.

^3^ NHC Key Laboratory of Pneumoconiosis, MOE Key Laboratory of Coal Environmental Pathogenicity and Prevention, Shanxi Key Laboratory of Chronic Respiratory Diseases and Pneumoconiosis, The First Hospital of Shanxi Medical University, Taiyuan, 030001, Shanxi, China.

^4^ Research Center for Reverse Microbial Etiology, Workstation of Academician, Shanxi Medical University, Taiyuan, People’s Republic of China.

^5^ Ophthalmic Microbiology Laboratory, Shanxi Eye Hospital, Shanxi Medical University, Taiyuan, Shanxi, P.R. China.

^6^ Department of Internal Medicine, The First Clinical Medical College, Shanxi Medical University, Taiyuan, 030001, Shanxi, China.

^7^ School of Public Health, Key Lab of Public Health Safety of the Ministry of Education and NHC Key Laboratory of Health Technology Assessment, Fudan University, Shanghai, China.

^8^ Department of Laboratory Medicine, First Hospital of Shanxi Medical University, 85 Jiefang South Road, Taiyuan, 030001, Shanxi, China.

^9^ Department of Respiratory and Critical Care Medicine, Shanxi Hospital of Traditional Chinese Medicine, Taiyuan, China.

^10^ Department of Respiratory and Critical Care Medicine, Shanxi Bethune Hospital, Shanxi Academy of Medical Sciences, Tongji Shanxi Hospital, Third Hospital of Shanxi Medical University, Taiyuan, 030032, China.

^11^ College of Pulmonary & Critical Care Medicine, Chinese PLA General Hospital, Beijing, 100091, People’s Republic of China.

^12^ Experimental Research Center & Molecular Diagnostic Center, Capital Center for Children’s Health, Capital Medical University, Capital Institute of Pediatrics, Beijing, 100020, People’s Republic of China.

^13^ Beijing Research Center for Respiratory Infectious Diseases, Beijing 100020, China.

^*^Correspondence: Prof. Yiwei Shi, shiyw@sxmu.edu.cn; Prof. Kun Xiao, 13716608331@163.com; Prof. Yi Wang, wildwolf0101@163.com; Prof. Zhenjun Li, lizhenjun@icdc.cn.

^#^Hongquan Chen, Xiaoling Wang, Xiang Liu, and Kui Dong contributed equally to this work.

**Figure S1 Distribution of clinical characteristics in the integrated dataset.** (A) Age distribution of patients, color-coded by disease conditions. (B) Age distribution of KPP patients stratified by sex (left) and disease severity (right). (C) Lymphocyte counts (left), monocyte counts (middle), and CRP levels (right) in KPP patients stratified by disease severity. Box plots represent the IQR with the median line; whiskers indicate 1.5 × IQR, and outliers are shown as points. KPP, *Klebsiella pneumoniae* pneumonia; MKPP, mild *K. pneumoniae* pneumonia; SKPP, severe KPP; IQR, interquartile range; CRP, C-reactive protein.

**Figure S2 Single-cell transcriptional profiling of peripheral blood mononuclear cells from 100 subjects, related to Figure 1.** (A, C, E) Percentage of mitochondrial transcripts per cell (A), distribution of UMI counts per cell (C), and gene counts per cell (E) across disease conditions. (B, D, F) Percentage of mitochondrial transcripts per cell (B), distribution of UMI counts per cell (D), and gene counts per cell (F) across individual samples. (G) Box plots showing the log_10_-transformed number of cells per sample. UMI, unique molecular identifier.

**Figure S3 Clustering quality and immune cell alterations in peripheral blood mononuclear cells from healthy donors and *Klebsiella pneumoniae* pneumonia patients, related to Figure 1.** (A) UMAP embedding of PBMCs before and after Harmony integration, illustrating the correction of sample batch effects across datasets. (B) UMAP visualization of PBMCs colored by major immune cell subtypes (left) and disease conditions (right). (C) Dot plot showing the expression of selected marker genes across major immune cell populations. (D) Donut charts illustrating compositional changes of the major cell subtypes across disease conditions. (E) *R_O/E_* values were calculated as observed cell counts divided by expected cell counts. Dot color indicates depletion or enrichment relative to the expected frequency, and dot size corresponds to the magnitude of the *R_O/E_* value. UMAP, Uniform Manifold Approximation and Projection; PBMCs, peripheral blood mononuclear cells; *R_O/E_*, ratio of observed to expected.

**Figure S4 Characterization of hyperinflammatory cell subtypes across disease severity groups, related to Figure 2.** (A) Box plots showing inflammatory scores (left) and cytokine scores (right) across disease conditions. (B) Box plots showing inflammatory scores (top) and cytokine scores (bottom) stratified by immune cell subtype. Orange vertical shading highlights the identified hyperinflammatory monocyte subtypes. (C) Box plots comparing the relative frequencies (percentage of total PBMCs) of hyperinflammatory (left) and non-inflammatory (right) cell populations across disease conditions. For all box plots, the center line indicates the median, the box limits represent the IQR, and whiskers extend to 1.5 × IQR. Statistical significance was assessed using the Kruskal–Wallis test on sample-level pseudobulk means, with Bonferroni correction for multiple comparisons. ns, *p* > 0.05; **p* < 0.05; ***p* < 0.01; ****p* < 0.001. PBMCs, peripheral blood mononuclear cells; IQR, interquartile range.

**Figure S5 Identification of hyperinflammatory subtypes associated with potential inflammatory responses in peripheral blood mononuclear cells, related to Figure 2.** Box plots showing inflammatory scores (top) and cytokine scores (bottom) across HC (*n* = 54), MKPP (*n* = 23), and SKPP (*n* = 23). For each donor, scores were calculated by aggregating single-cell expression values within each immune cell subtype. The center line indicates the median, boxes represent the IQR, and whiskers extend to 1.5 × IQR. Statistical significance was assessed using the Kruskal–Wallis test on sample-level pseudobulk means, with Bonferroni correction for multiple comparisons. ns, *p* > 0.05; **p* < 0.05; ***p* < 0.01; ****p* < 0.001. HC, healthy controls; MKPP, mild *Klebsiella pneumoniae* pneumonia; SKPP, severe *K. pneumoniae* pneumonia; IQR, interquartile range.

**Figure S6 Characterization of hyperinflammatory subtypes associated with potential inflammatory responses in peripheral blood mononuclear cells, related to Figure 2.** (A) Dot plot showing the expression of selected inflammatory genes across all cell subtypes. Dot size represents the fraction of cells expressing each gene, and color intensity indicates the average expression level. (B) Pie charts showing the relative contribution of each of the ten hyperinflammatory immune cell subtypes to the total *S100A8/A9/A12* score calculated from all cells in SKPP cases. (C) Box plots showing pseudobulk expression levels of *S100A8*, *S100A9*, and *S100A12* derived from scRNA-seq data across disease conditions. The center line indicates the median, boxes represent the IQR, whiskers extend to 1.5 × IQR, and outliers are plotted individually. (D) Exploratory patient-level correlations of inflammatory, cytokine, and *S100A8/A9/A12* scores with plasma CRP and PCT concentrations in pooled patients with MKPP and SKPP. Each point represents one independent patient. The number of patients with available paired measurements, Spearman’s ρ, bootstrap 95% confidence interval, nominal *p* value, and Benjamini–Hochberg FDR-adjusted *p* value are shown. FDR correction was performed across the six prespecified score–marker correlations. Lines and shaded areas represent linear fits and 95% confidence intervals for visualization only. (E) Bar plots showing the relative contribution of individual immune cell subtypes to the *TLR4* (top) and *MYD88* (bottom) signaling scores. (F) Heatmap showing the number of CellPhoneDB-predicted ligand–receptor associations among hyperinflammatory subtypes in pooled SKPP cells. Statistical significance was assessed using the Kruskal–Wallis test on sample-level pseudobulk means, with Bonferroni correction for multiple comparisons. ns, *p* > 0.05; **p* < 0.05; ***p* < 0.01; ****p* < 0.001. SKPP, severe *Klebsiella pneumoniae* pneumonia; CRP, C-reactive protein; PCT, procalcitonin; IQR, interquartile range.

**Figure S7 Extended characterization of CD4⁺ T cell states across disease conditions, related to Figure 3.** (A) Heatmap showing the expression of representative marker genes across the nine annotated CD4⁺ T cell subtypes. (B) PAGA-based topology analysis of CD4⁺ T cell subtype connectivity. Node size reflects relative abundance, and edge width indicates connectivity strength. (C) Heatmap showing functional signature scores across CD4⁺ T cell subtypes. (D) Stacked bar plots showing the relative proportions of CD4⁺ T cell subtypes across disease conditions. (E) Heatmap showing regulon activity (Z-score) of representative transcription factors associated with CD4T_07_Th1 cells across disease conditions. (F) Bar plot showing the frequency of CD4T_08_Treg cells across disease conditions (left), and heatmap showing the expression of representative immunoregulatory molecules in CD4T_08_Treg cells (right). (G) Bar plot showing the frequency of CD4T_09_Pro cells across disease conditions. (H) Bar plots showing GSEA of Hallmark pathways in CD4T_09_Pro cells. Top panel compares MKPP versus HC, and bottom panel compares SKPP versus HC. Bar length indicates −log_10_(FDR q-value). (I) Mfuzz clustering of differentially expressed genes in CD4T_09_Pro cells, showing dynamic expression patterns across disease conditions. (J) Bar plot showing GO enrichment analysis of Cluster 4 genes in CD4T_09_Pro cells. Enriched GO terms are grouped into functional categories, including cell cycle, spindle, and DNA repair. Bar length indicates −log_10_(adjusted *p* value), and representative genes contributing to each GO term are shown below the corresponding bars. Statistical significance was assessed using the Kruskal–Wallis test on sample-level pseudobulk means, with Bonferroni correction for multiple comparisons. ns, *p* > 0.05; **p* < 0.05; ***p* < 0.01; ****p* < 0.001. HC, healthy controls; MKPP, mild *Klebsiella pneumoniae* pneumonia; SKPP, severe *K. pneumoniae* pneumonia; PAGA, partition-based graph abstraction; GSEA, Gene Set Enrichment Analysis; FDR, false discovery rate; GO, Gene Ontology.

**Figure S8 Flow cytometry plots showing gating strategy in CD4⁺ T cells from patients with severe *Klebsiella pneumoniae* pneumonia, related to Figure 3.** Flow cytometry plots showing gating strategy and representative exhaustion-associated molecules in CD4⁺ T cells from 8 patients (SKPP05-SKPP06, SKPP08, SKPP10-SKPP14) with SKPP. SKPP, severe *K. pneumoniae* pneumonia.

**Figure S9 Extended characterization of CD8⁺ T cell states across disease conditions, related to Figure 4.** (A) Violin plot showing the expression of selected marker genes across CD8⁺ T cell subtypes. (B) Box plots showing exhaustion scores (top) and cytotoxicity scores (bottom) across CD8⁺ T cell subtypes. (C) Dot plot showing the expression of exhaustion-related genes across disease conditions. Dot size represents the fraction of cells expressing each gene, and color intensity indicates the average expression level. (D) Dot plot showing the expression of cytotoxicity-related genes across CD8⁺ T cell subtypes. (E) Box plot showing apoptosis scores across CD8⁺ T cell subtypes. (F) Box plots showing the expression of apoptosis-associated genes across disease conditions. (G) GSEA plots showing enrichment of Hallmark pathways in CD8⁺ T cells for the MKPP versus HC comparison. Enriched pathways are shown as barcode plots, with dot size indicating gene-set size, dot color indicating NES, and the heatmap-like bar indicating −log_10_(adjusted *p* value). Statistical significance was assessed using the Kruskal–Wallis test on sample-level pseudobulk means, with Bonferroni correction for multiple comparisons. ns, *p* > 0.05; **p* < 0.05; ***p* < 0.01; ****p* < 0.001. HC, healthy controls; MKPP, mild *Klebsiella pneumoniae* pneumonia; SKPP, severe *K. pneumoniae* pneumonia; GSEA, Gene Set Enrichment Analysis; NES, normalized enrichment score.

**Figure S10 Flow cytometry plots showing gating strategy in CD8⁺ T cells from patients with severe *Klebsiella pneumoniae* pneumonia, related to Figure 4.** Flow cytometry plots showing gating strategy and representative exhaustion-associated molecules in CD8⁺ T cells from 8 patients (SKPP05-SKPP06, SKPP08, SKPP10-SKPP14) with SKPP. SKPP, severe *K. pneumoniae* pneumonia.

**Figure S11 Extended characterization of B cell states across disease conditions, related to Figure 5.** (A) Stacked violin plot showing the expression of selected marker genes across B cell subtypes. (B) Patient-level correlation between plasma cell and Tfh cell counts in SKPP. Each point represents one patient. Spearman’s *ρ*, bootstrap 95% CI, nominal *p* value, and BH-FDR-adjusted *p* value are shown. FDR correction was performed across the prespecified MKPP and SKPP correlations. (C) UMAP showing pseudotime distribution of B cells across disease conditions. (D) Dot plots showing the expression of selected activation- and BCR signaling-related genes in B_01_Naive and B_03_Memory cells across disease conditions. Dot size represents the fraction of cells expressing each gene, and color intensity indicates the average expression level. SKPP, severe *Klebsiella pneumoniae* pneumonia; UMAP, Uniform Manifold Approximation and Projection; BCR, B cell receptor.

**Figure S12 Extended characterization of myeloid cell states across disease conditions, related to Figure 6.** (A) Heatmap showing the expression of selected canonical marker genes across myeloid cell subtypes. (B) Stacked area plot showing the relative proportions of myeloid cell subtypes across disease conditions. (C) Dot plots showing the expression of selected MHC class II genes in DC_01_mDC cells across disease conditions. Dot size represents the fraction of cells expressing each gene, and color intensity indicates the average expression level. (D) Dot plot showing predicted ligand–receptor interactions between DC_01_mDC cells and naïve CD4⁺ T cell subtypes. (E) Dot plot showing selected monocyte- and mMDSC-associated genes across myeloid cell subtypes. (F) Scatter plots showing associations between mMDSC immunoregulatory scores and functional states of CD4_Th1 and CD4_Treg cells. Each point represents one donor. Spearman’s ρ and Benjamini–Hochberg FDR-adjusted *p* values are shown. (G) Raincloud plot showing mMDSC immunoregulatory scores across disease conditions. (H) Dot plot showing selected predicted ligand–receptor interactions between monocytic MDSCs and T cell subtypes in MKPP and SKPP groups. (I) Bar plot showing GO terms enriched among genes upregulated in MKPP-associated monocytic MDSCs. Bar length indicates −log_10_(adjusted *p* value). (J) Venn diagram showing the overlap of upregulated DEGs in classical monocytes from MKPP versus HC and SKPP versus HC comparisons. Representative MKPP-specific and SKPP-specific genes are shown beside the diagram. (K) GO enrichment analysis of MKPP-specific and SKPP-specific genes in classical monocytes. Blue terms represent pathways enriched among MKPP-upregulated genes, and red terms represent pathways enriched among SKPP-upregulated genes. Numbers above bars indicate the number of genes contributing to each GO term. Statistical significance was assessed using the Kruskal–Wallis test on sample-level pseudobulk means, with Bonferroni correction for multiple comparisons. ns, *p* > 0.05; **p* < 0.05; ***p* < 0.01; ****p* < 0.001. HC, healthy controls; MKPP, mild *Klebsiella pneumoniae* pneumonia; SKPP, severe *K. pneumoniae* pneumonia; mMDSC, monocytic myeloid-derived suppressor cell; MHC, major histocompatibility complex; GO, Gene Ontology; DEGs, differentially expressed genes.

**Figure S13 Bar chart showing the composition of *C1QA/B/C* scores, broken down by relative contributions from each cell subtype.**
